# Long-read sequencing reveals genomic structural variations that underlie creation of quality protein maize

**DOI:** 10.1038/s41467-019-14023-2

**Published:** 2020-01-07

**Authors:** Changsheng Li, Xiaoli Xiang, Yongcai Huang, Yong Zhou, Dong An, Jiaqiang Dong, Chenxi Zhao, Hongjun Liu, Yubin Li, Qiong Wang, Chunguang Du, Joachim Messing, Brian A. Larkins, Yongrui Wu, Wenqin Wang

**Affiliations:** 1 School of Agriculture and Biology, Shanghai Jiao Tong University, Shanghai, 200240 China; 2 National Key Laboratory of Plant Molecular Genetics, CAS Center for Excellence in Molecular Plant Sciences, Institute of Plant Physiology & Ecology, Shanghai Institutes for Biological Sciences, Chinese Academy of Sciences, Shanghai, 200032 China; 3 Institute of Biotechnology and Nuclear Technology, Sichuan Academy of Agricultural Sciences, Chengdu, 610061 China; 4 University of the Chinese Academy of Sciences, Beijing, 100049 China; 5 Waksman Institute of Microbiology, Rutgers University, 190 Frelinghuysen Road, Piscataway, NJ 08854 USA; 6 BGI Education Center, University of Chinese Academy of Sciences, Shenzhen, 518083 China; 7 State Key Laboratory of Crop Biology, College of Life Sciences, Shandong Agricultural University, Tai’an, 271018 China; 8 College of Agronomy, Qingdao Agricultural University, Qingdao, 266109 China; 9 Department of Biology, Montclair State University, Montclair, NJ 07043 USA; 10 School of Plant Sciences, University of Arizona, Tucson, AZ 85721 USA

**Keywords:** Agricultural genetics, Genomics, Natural variation in plants, Plant breeding

## Abstract

Mutation of *o2* doubles maize endosperm lysine content, but it causes an inferior kernel phenotype. Developing quality protein maize (QPM) by introgressing *o2 modifier*s (*Mo2*s) into the *o2* mutant benefits millions of people in developing countries where maize is a primary protein source. Here, we report genome sequence and annotation of a South African QPM line K0326Y, which is assembled from single-molecule, real-time shotgun sequencing reads collinear with an optical map. We achieve a N50 contig length of 7.7 million bases (Mb) directly from long-read assembly, compared to those of 1.04 Mb for B73 and 1.48 Mb for Mo17. To characterize *Mo2*s, we map QTLs to chromosomes 1, 6, 7, and 9 using an F_2_ population derived from crossing K0326Y and W64A*o2*. RNA-seq analysis of QPM and *o2* endosperms reveals a group of differentially expressed genes that coincide with *Mo2* QTLs, suggesting a potential role in vitreous endosperm formation.

## Abstract (web summary)

The South African quality protein maize (QPM) cultivars have the desired high lysine content and kernel hardness due to o2 mutation and the introgression of modifiers of o2 (Mo2) QTLs, respectively. Here, the authors assemble the genome of a QPM line and identify candidate genes underlying Mo2 QTLs.

## Introduction

Shotgun DNA sequencing has been used to build contiguous sequence information of entire chromosomes^1^. However, previous sequencing reads were too short to resolve the order of repetitive sequences, because the repeats were longer than an individual read, or sequence variations between two tandem copies were not of sufficient length to distinguish them. A recent platform with long reads from single DNA molecules can overcome the low resolution of reconstructed repetitive regions in complex plant genomes, which allows researchers to produce a genome with the fewest gaps of any previously sequenced maize genome and a high-resolution sequence analysis demonstrating allelic diversity.

**Fig. 1:**
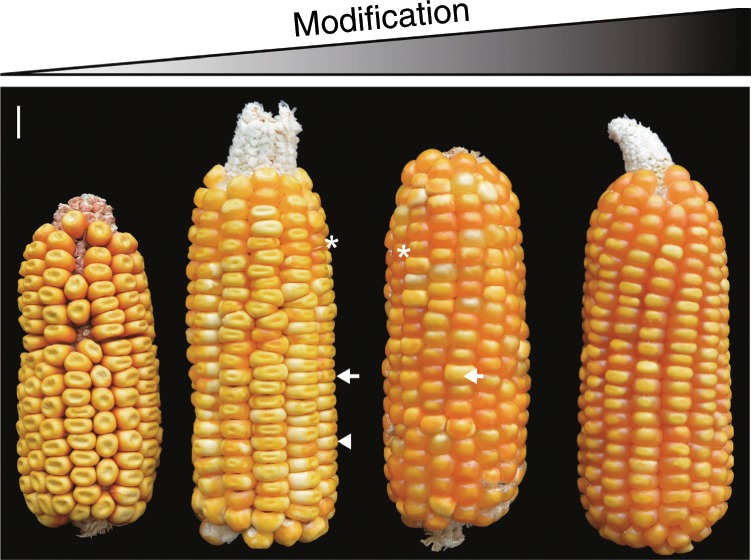
Segregation of endosperm modification in QPM Pool 42. Because QPM Pool 42 is not homozygous for *o2* modifiers, it segregates progeny ears and kernels with varying degrees of modification. From left to right, ear 1 is totally unmodified and therefore all kernels are opaque; ear 2 is partially modified and only a few kernels are vitreous; ear 3 is mostly modified, with sporadic kernels that are mosaics of virtuousness and opaque endosperm; ear 4 is almost completely modified. The asterisk, arrow and arrowhead in ears 2 and 3 indicate kernels with vitreous, mosaic and opaque phenotypes. Scale bar = 10 mm.

It is well known that the most abundant storage proteins in maize endosperm, prolamins called zeins, are deficient in the essential amino acids, lysine and tryptophan, requiring costly dietary supplementation. A natural maize high-lysine variant lacks a functional Opaque2 (O2) bZIP transcription factor that regulates expression of most zein genes. In the *o2* mutant, the amount of zein proteins is dramatically reduced, but is compensated by an increase of other proteins (non-zeins), thereby doubling the lysine levels of total protein compared to normal maize^2^. However, the chalky and soft endosperm texture of *o2* impacts the transport and storage of the grain, and facilitates fungal infection and insect susceptibility^2^. To circumvent these problems, a long-term effort by plant breeders restored kernel hardness in the *o2* mutant via the introduction of quantitative trait loci (QTLs) known as *o2 modifier*s (*Mo2*s), creating quality protein maize (QPM)^2^ (Fig. 1). QPM is now produced in many developing countries, where people consume maize as a regular protein source.

There is good evidence that the vitreous kernel phenotype of QPM is determined by several unlinked loci^3^. The challenge for maize breeders was to simultaneously introduce multiple non-linked *Mo2*s from tropical germplasms into temperate inbred lines, while retaining a homozygous recessive *o2* locus^3,4^. It was shown that the 27-kD γ-zein (γ27), a cysteine-rich protein, is an important factor for endosperm modification in QPM^5^. This protein plays a role in the initiation and stabilization of zein protein bodies (PBs), allowing them to accumulate in the rough endoplasmic reticulum^6,7^. *qγ27*, a major *Mo2* derived from a gene duplication at the *γ27* locus, elevates both 27-kD γ-zein transcript and protein levels, leading to more PBs that that coalesce around starch granules; this creates vitreous endosperm during kernel desiccation^3,5,8^. However, other mechanisms contributing to how starchy *o2* endosperm is modified in QPM are not well understood.

To characterize additional *Mo2* loci, we sequence the genome of a QPM line K0326Y and compare it with B73 and Mo17 for genomic variations. We conduct a bulked segregation analysis of the F_2_ population of K0326Y and W64A*o2* and identify *Mo2* QTLs. We describe a set of differentially expressed genes (DEGs) from RNA-seq analysis between QPMs and *o2* mutants. We emphasize a group of candidate genes tightly linked to *Mo2* loci, with genomic structural variations and altered expression in QPMs. Based on these and previous studies, we hypothesize that an increased generation of ATP through glycolysis and an enhanced unfolded protein response (UPR) can contribute to alleviating an energy deficiency in *o2*, and directly or indirectly facilitate the formation of vitreous endosperm in QPM endosperm. The high-quality QPM genome sequence and the underlying mechanism of vitreous endosperm development are useful for future molecular characterization and creation of QPMs.

## Results

### Genome assembly and validation

The genome of K0326Y was sequenced and assembled using three technologies: PacBio single-molecule real-time (SMRT) sequencing, Illumina paired-end sequencing and BioNano optical mapping (Methods). The initial genome of K0326Y was assembled into 2,148 Mb by using 28.35 million long reads with an N50 read length of 16.6 kb and about 139-fold coverage (Supplementary Tables 1–3 and Supplementary Fig. 1), resulting in 1,221 contigs with an N50 of 6.99 Mb (Table 1, Supplementary Tables 4,  5), comparable to the recent published SK genome with a contig N50 of 5.93 Mb (ref. ^9)^. Assembled contigs were corrected with 132.5 Gb PacBio consensus sequences and 217.5 Gb high-quality Illumina paired-end reads. These processed contigs were subjected to hybrid assembly by optical maps created from 389.3 Gb BioNano molecules. The assembly contained 870 scaffolds with an N50 scaffold size of 27.98 Mb (Table 1 and Supplementary Table 5). The B73 reference genome^10^ was used to map and orient scaffolds onto K0326Y chromosomes, which accounted for 97.74% (2,112 Mb) of the total assembled sequences (Supplementary Table 6). The total assembly size of the K0326Y genome was 2,161 Mb, similar to the recently published B73 (2,106 Mb)^10^ and Mo17 (2,183 Mb)^11^. However, K0326Y had only 438 gaps and a final N50 contig size of 7.77 Mb, providing 5-fold higher contiguous sequences than B73 (contig N50: 1.25 Mb) and Mo17 (contig N50: 1.47 Mb) (Table 1 and Supplementary Fig. 2).

**Table 1:** Global statistics for the K0326Y genome assembly.

	PacBio assembly	BioNano assembly	Hybrid assembly	Pseudomolecule	Final assembly
Assembled size (Mb)	2,148	2,147	2,162	2,112	2,161
Contig N50 (Mb)	6.99	1.43	7.77	7.99	7.77
Scaffold N50 (Mb)	–	–	27.98	–	22.78
Longest scaffold (Mb)	35.54	7.29	69.25	–	–
Scaffold number	1,221	2,191	870	10	673

To evaluate the quality of the assembled K0326Y genome, a high-density maize pan-genome genetic map containing ~4.4 million genotype-by-sequencing (GBS) tags^12^ was used. The alignment of the K0326Y genome with anchored GBS tags showed high consistency with respect to the position and orientation of the mapped scaffolds (Supplementary Fig. 3). The assembly accuracy and completeness were supported by 100-fold Illumina reads with 93.8% mapping rate (Supplementary Table 2). Approximately 95.8% (1380 out of 1440 genes) of embryophyta genes were detected in the K0326Y assembly according to BUSCO^13^, a percentage similar to that for B73 (96.1%) and Mo17 (95.4%) genome (Supplementary Table 7).

### Repeat analysis

**Fig. 2:**
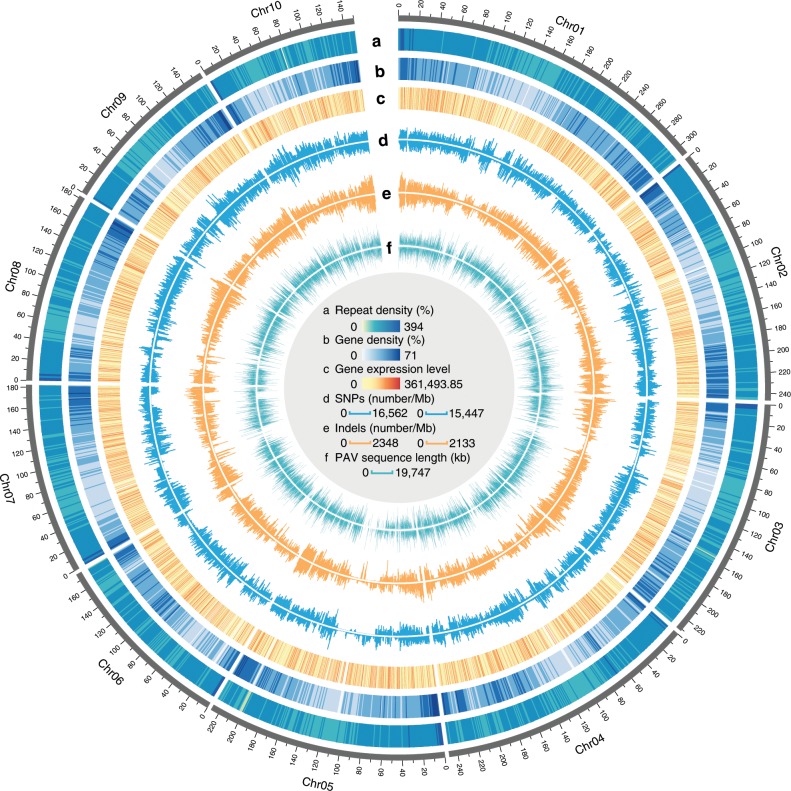
Genomic landscape of the K0326Y genome. **a** Transposable-element density. **b** gene density. **c** Gene expression levels. **d** SNPs. **e** Indels. **f** PAV distribution. For the tracks of **d**, **e**, and f, the outer layer is B73 and the inner is Mo17. The sliding window is 1 Mb for all tracks. Source data are provided as a Source Data file.

Uninterrupted sequences represent a major improvement for chromosomal regions with high contents of repeat sequences^10^ (Fig. 2). A total of 83.32% of the K0326Y genome consisted of repetitive elements, including retrotransposons (77.38%), DNA transposons (4.72%), and unclassified elements (0.49%) (Supplementary Table 8). From retrotransposons, 136,191 high-confidence intact long terminal repeats (LTRs) were identified within the 10 chromosomes, slightly more than that reported for B73 (ref. ^10^). Families of *Gypsy* and *Copia* retrotransposons represented ~43.44% and 23.74%, respectively, of the K0326Y assembled sequences. The overall composition of the retrotransposon families in K0326Y was very similar to that in the Mo17 and B73 genomes, as they occurred before domestication (Supplementary Fig. 4). Still, a few families showed copy number variations among K0326Y, B73, and Mo17, such as the LTR family of *Ty1*/*Copia RLC_ebel*, with five copies in B73, but 159 and 154 copies in K0326Y and Mo17. There were 647 copies of *Ty3/Gypsy RLG_huck_*AC214833 in B73, compared with 482 and 446 copies in K0326Y and Mo17, respectively. A more similar pattern of LTR family copy numbers was observed in K03236Y and Mo17, compared to K03236Y and B73. The centromeric regions for each chromosome were reconstructed by analysis of centromere-related long terminal repeat (CRM) (Supplementary Table 9) and 156-bp tandem repeats (CentC)^14^ (Supplementary Table 10), showing a similar distribution pattern like B73 (Supplementary Figs. 5, 6).

### Genome annotation

A total of 1,618,691 high-quality full-length non-chimeric reads (HQ-FLNC), i.e., full-length cDNA consensus sequences, were identified from PacBio isoform sequencing, generating 247,616 non-redundant transcripts to predict gene models using marker-P^10,15^ (Supplementary Table 11). A set of 38,238 genes with 60,475 transcripts was identified in the K0326Y genome (Supplementary Table 11), comparable to B73 (39,200 genes)^10^ and Mo17 (38,620 genes)^11^. The full-length cDNA data improved the K0326Y genome annotation by the fact that 69% of gene models were supported by full-length transcripts (CDS coverage > 50%) (Supplementary Table 12). In addition, 37,861 (99.01%) K0326Y gene models could be assigned to specific chromosomal locations.

### Comparative genomics

Understanding the intraspecific variation of maize has important implications for crop improvement and plant breeding. Given the contiguous genome sequences, we were able to investigate structural diversity between the tropical K0326Y and two temperate inbreds, B73 (ref. ^10^) and Mo17 (ref. ^11)^. Approximately 58% of K0326Y genome sequences matched one-to-one syntenic blocks of B73 and Mo17, respectively (Supplementary Fig. 6). The genome-wide proportion of syntenic regions between K0326Y and B73 or Mo17 was lower than a recent analysis between B73 and Mo17 (ref. ^11^), possibly due to the intensified divergent selections of seed yield and reproductive success between B73 and Mo17, which is important for heterosis between the two heterotic groups^16^. There were two inversions found to be specific for K0326Y compared to temperate and tropical maize lines. Chromosome 1 harbored one large pericentric inversion of 8.5 Mb, which was supported by the BioNano optical map with their breakpoints (Supplementary Figs. 6, 7). The inversion was different, with one of 1.7-Mb of the SK Chromosome 1 that originated from South America, indicating that this structural variation could be specific for K0326Y^9^. It was reported that some genes in the inverted region exhibited a significant association with maize flowering time^17^. Another large paracentric inversion of 5.8 Mb was located at the centromere of chromosome 4 (Supplementary Figs. 6, 7). Most genes in the inversion have functions related to carbohydrate metabolism and gene regulation. K0326Y is a QPM line carrying the *o2* mutation. Comparison of the *O2* gene in K0326Y with B73 and Mo17 indicated there was a 4958-bp *rbg* transposon insertion 249-bp upstream of the start codon (ATG), similar to other *o2* alleles (Supplementary Fig. 8)^18^. As expected, gene expression data showed that transcription of *O2* was inhibited due to the *rbg* insertion.

### Genomic polymorphism and structural variations

The maize genome exhibits very high levels of genetic diversity with respect to SNPs, small InDels, and structural variations, which contribute to phenotypic diversity and heterosis in maize hybrids^19^. We identified a total of 10,205,511 SNPs and 1,397,901 InDels (<100 bp) between K0326Y and B73, with an average of 8.35 SNPs and 1.14 InDels per kilobase. There were 9655,364 SNPs and 1,458,329 InDels (<100 bp) between K0326Y and Mo17, with an average of 7.77 SNPs and 1.17 InDels per kilobase (Supplementary Table 13). The genetic polymorphism affects 8,702 genes in B73 and 6,009 genes in Mo17, including frameshift, stop-codon loss and stop-codon acquisition (Supplementary Fig. 9), which could contribute to other functions in K0326Y. We identified 19,778 insertions (>100 bp) and 39,931 deletions between K0326Y and B73 that could affect 6,538 and 10,463 genes, respectively. In the case of K0326Y and Mo17, 32,071 insertions could affect 9,323 genes and 46,381 deletions were found in 12,456 genes, respectively (Supplementary Tables 14, 15).

The distribution of presence/absence variations (PAVs) that were only present in K0326Y but entirely missing in B73 and Mo17 was identified. A total of 39,479 segments were identified in K0326Y with a total length of 154.7 Mb absent in B73. Similarly, there were 37,906 segments in K0326Y with a total length of 149.5 Mb missing in Mo17 (Fig. 2). These PAV regions affected 3568 genes in K0326Y (Supplementary Data 1). The expression of 631 PAV genes was upregulated based on comparison of RNA-seq data from 16-DAP developing endosperm of K0326Y QPM compared to W64A*o2*. These genes were enriched for pathways like starch biosynthesis and metabolic processes, ATPase activity, auxin biosynthesis and sulfur transmembrane activity (Supplementary Fig. 10). Interestingly, it was found that the PAV genes in B73, Mo17, and PH207 are also present in wild maize relatives, and occurred before the completion of maize domestication, but after the divergence of sorghum and maize^11^.

Gene duplications provide a mechanism to change phenotypes via a gene dosage effect or a gene function from divergence. We found that the number of singleton genes in B73, Mo17, and K0326Y were quite conserved, whereas K0326Y had fewer segmental duplications (12,259), but more dispersed duplicated genes (16,777) compared to B73 and Mo17 (Supplementary Table 16). Tandemly duplicated genes were located in proximal neighborhoods and potentially shared the same regulatory elements. We identified 1261 tandem gene copy clusters, accounting for 3842 annotated genes in the K0326Y genome (Supplementary Table 16).

### Genetic mapping of *Mo2*s

**Fig. 3:**
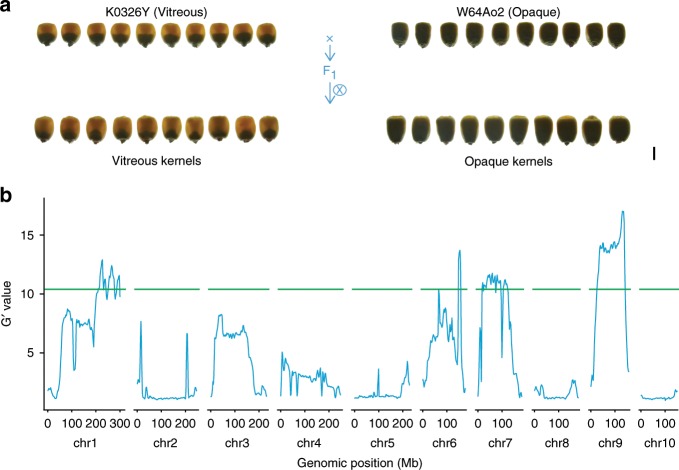
Mapping of *o2* modifiers. **a** Construction of F_2_ population by using K0326Y(QPM) and W64A*o2* (*o2*). K0326Y has a vitreous phenotype and W64A*o2* has an opaque phenotype. **b** mapping o2 modifiers by bulk segregant analysis (BSA) of a segregating population derived from a K0326Y and W64A*o2* cross. The G’ value is a smoothed version of the standard G statistic in each 4-Mb sliding window. Green line indicates threshold of G’ value corresponding to an FDR of 8 × 10^−7^. Scale bar = 5 mm.

Given the ubiquitous genetic variability between QPM and other *o2* mutants, it was expected that certain variations, including the *Mo2* genes, are responsible for their phenotypic differences. To map chromosomal regions linked with endosperm modification, we followed the methods of Holding^3,4^ and crossed K0326Y with W64A*o2*. The pooled DNA from leaf tissue of F_2_ vitreous and opaque kernels was used for a bulked segregation analysis, with next-generation sequencing (BSA-seq) to identify QTLs associated with endosperm modification. To avoid a masking effect by the major *Mo2* (*qγ27*), we selected vitreous and opaque kernels with two copies of *γ27* gene at the *γ27* locus for QTL-seq analysis^8^ (Supplementary Fig. 11). Three *Mo2* QTLs were located on chromosomes 1, 7, and 9 (Fig. 3b and Supplementary Fig. 12), consistent with previous mapping data^3,4^. Another QTL with a sharp peak was found at a region on chromosome 6, with a large K0326Y-specific fragment insertion (Supplementary Fig. 3). Whether it is a false-positive or not needs further examination, because crossovers in a F_2_ population are very limited.

To identify candidate genes corresponding to *Mo2* QTLs, total RNA from developing maize endosperm was collected at 16 DAP from vitreous and opaque kernels. The transcriptomic profiles were built to compare gene expression levels between K0326Y and W64A*o2* and between CM105*Mo2* (another QPM line in the CM105 background) and CM105*o2*. There were 1791 DEGs that overlapped in the two comparisons, of which 926 were upregulated and 865 downregulated (Supplementary Data 2). The increased DEGs were significantly enriched in the terms of chaperone binding, unfolded protein binding, protein folding, and response to heat and temperature stimulus (Supplementary Fig. 13). Notably, 43 genes encoding heat shock protein (HSPs) and HSP transcription factors were significantly upregulated in the QPM (Supplementary Data 3). As previously suggested^20^, they might be activated and reduce stress effects by reassembling unfolded or aggregated proteins created by the *o2* mutation.

There were 216 (117 upregulated and 99 downregulated) DEGs that cross-checked with genes from the QTL-seq analysis, narrowing the pool of candidate genes contributing to a vitreous kernel phenotype and environmental adaption (Supplementary Data 4). After sorting the 216 gene list, there were 125 containing SNPs, 44 with insertions (>100 bp), and 43 with deletions (>100 bp), compared to B73, as well as 18 exhibiting tandem duplications, and 19 belonging to PAVs (Supplementary Data 4). Still, most DEGs that could not be mapped to QTLs might not necessarily be causative of the QPM phenotype, but they can provide clues as to an effect of Mo2s that set off a signaling cascade, leading to formation of vitreous endosperm. They could also be under regulation of Mo2s, reflecting downstream effects of changes in kernel composition.

As we integrated genome variations to identify candidate genes associated with *Mo2*s that deserve further study, those with allelic variations, elevated expression, and located within QTL regions became apparent. On chromosome 1, there was 85-bp deleted from the *O10* promoter in K0326Y, which was highly expressed in K0326Y and CM105*Mo2* QPM lines*. O10* encodes a cereal-specific protein that regulates zein deposition and organization in PBs^21^. Mutations in *O10* create abnormal distribution of zeins in PBs. Whether upregulation of *O10* functions as a *Mo2* or is required for *Mo2*-mediated endosperm modification remains to be investigated.

Tandem duplication of the *γ27* locus (*qγ27*), which was previously designated the *Standard* allele (*S*) for bearing two copies of *γ27* gene, is a major *Mo2* for endosperm modification in QPM^8^. Besides *γ27*, this duplication involves three other genes (GRMZM2G565441, GRMZM2G138976, and GRMZM5G873335) based on B73_vs3, which were merged into one gene with fifteen exons encoding ARID-transcription factor 4 (Zm00001d020593)^10^ in B73_vs4 and K0326Y. Although all QPM lines have this duplication, a contiguous genomic sequence was not previously available and its variation was unknown^8^. Here, we were able to expand this sequence to 28 kb, totally covering the duplication (~15 kb). The duplicated fragments have many SNPs that could distinguish them. The first copy of *ARID4* has a 1923-bp deletion in its 3’ end, resulting in four missing exons, compared to the second gene copy (Supplementary Fig. 14). A polymorphic PCR primer pair flanking this deletion could be designed to select for the duplication during QPM breeding, depending on the presence of two PCR bands. A global alignment showed that the *qγ27* locus between Mo17 and K0326Y were nearly identical. The B73-*γ27* allele was closer to the first fragment copy in K0326Y, and B73-*ARID4* was closer to the second one in K0326Y (Supplementary Fig. 15). This indicates that the single-copy *γ27* allele in B73 could have been produced from DNA rearrangement of the duplicated allele. Therefore, modern maize lines might have had at least three types of single-copy *γ27* allele, whereas the tandem duplication might have occurred early before maize domestication (Supplementary Fig. 16).

**Fig. 4:**
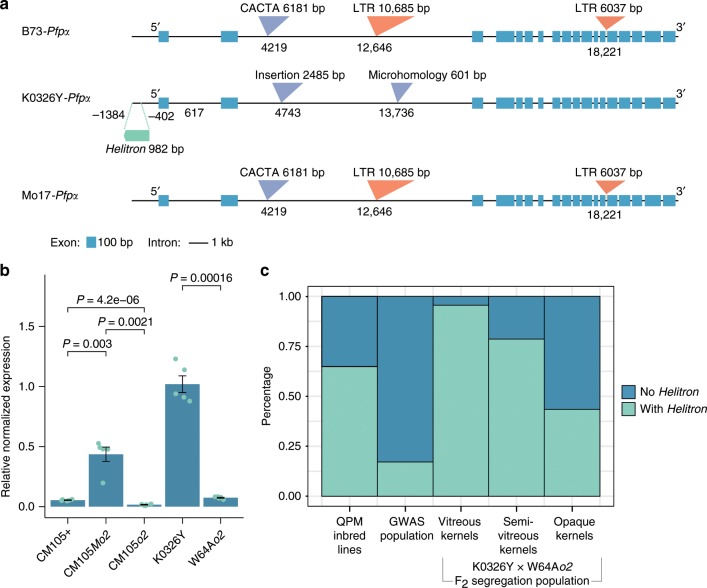
*Pfpα* associated with *o2* endosperm modification. **a** Structural variation of *Pfpα* among K0326Y, Mo17, and B73. The gene structure of *Pfpα* is shown with exons in blue boxes and introns with a black line. The *Helitron* in the promoter is labelled as a green bar. The LTR retrotransposon insertions, abbreviated with LTR, are illustrated by orange inverted triangles. Other insertions are represented by purple inverted triangles. The physical location is based on the K0326Y genome. (**b**) Relative gene expression of *Pfpα* for wild-type (CM105+), *o2* (CM105*o2* and W64A*o2*), and QPM (CM105M*o2* and K0326Y). *P*-values are determined by two-tailed Student’s *t*-test. The error bars are defined as sample standard deviation from the sample size (biologically independent samples) of *n* = 5. **c** Distribution of *Helitron* insertions in QPM inbred lines, GWAS population and K0326Y X W64A*o2* F_2_ segregation population; *x*-axis shows the maize populations and the *y*-axis is the linked percentage. Source data underlying Figs. 4b, c are provided as a Source Data file.

In agreement with previous studies, our analysis of QTL mapping (BSA-seq) and gene expression (RNA-seq) identified another promising *Mo2* candidate gene on chromosome 9, *Pfpα*^3,4^. *Pfpα* encodes the α-regulatory subunit of pyrophosphate-dependent fructose-6-phosphate 1-phosphotransferase and serves as a non-ATP-requiring enzyme during glycolysis. Transcript and protein levels, as well as enzyme activity of *Pfpα* were greatly increased in QPM endosperms relative to normal and *o2* mutants^3,4,20^. As noted, this could lead to increased glycolytic flux in QPM endosperm and ameliorate an aspect of the *o2* phenotype including a limitation in ATP^20^. The identity of the causative polymorphisms and underlying expression mechanisms influencing *Pfpα* had not been elucidated due to the lack of a QPM genome sequence. We found that the *Pfpα* locus in K0326Y showed dramatic structural variations compared to Mo17 and B73. In K0326Y*-Pfpα*, there was a 983-bp *Helitron* in the promotor and a 2485-bp insertion in the second intron, compared to Mo17-*Pfpα* and B73*-Pfpα*. However, the Mo17-*Pfpα* and B73*-Pfpα* both contained a 6181-bp CACTA, a 10,685-bp retrotransposon in the second intron, and a 6037-bp retrotransposon in the 13th intron, compared to the K0326Y*-Pfpα* (Fig. 4a). To investigate whether these insertions and deletions affect *Pfpα* expression, the transcripts of each exon were normalized to the exon size and sequencing depth. We found that the transcript abundance of the whole gene and each exon of *Pfpα* in QPMs (K0326Y and CM105*Mo2*) were higher than non-QPM lines (W64A*o2*, CM105*o2*, CM105+) (Fig. 4b and Supplementary Fig. 17). In addition, we found that 65% of QPM lines have the *Helitron* insertion. The *Helitron* insertion was retained in 95% of the vitreous endosperm kernels of the segregating F_2_ population (Fig. 4c), and is therefore clearly associated with the vitreous kernel phenotype. Mobile genetic elements can drive genome evolution, and also alter gene expression by insertion within introns, exons or regulatory region. Transposon-mediated mutations in the promoter of *Adh1* resulted in a significant increase of gene expression in pollen^22^, and a transposable element inserted into the regulatory region of a maize domestication gene (*teosinte branched1*, *tb1*) acted as an enhancer of its expression, which partially explained increased apical dominance in maize^23^. The *Helitron* insertion into the promoter of *Pfpα* might be partially causative of endosperm modification in QPM. Whether other transposable elements influence functional variation requires further research.

Among the *Mo2* candidates, two genes, *SR45a* and *ERDJ3A*, were extremely close to the peak on chromosome 9 (Supplementary Data 4). The level of the *SR45a* transcripts increased 2–28 times in QPM compared with *o2* mutants (Supplementary Fig. 18 and Supplementary Data 4). This gene was responsible for RNA splicing, and was also elevated when Arabidopsis was under stress^24^. Interestingly, there was a 399-bp DNA transposon of the *hAT* element in the 5th intron of *SR45a* compared with B73 and Mo17, and this region, with an associated index of 61% (Supplementary Fig. 18), was highly linked to the vitreous trait in the F_2_ population. It was reported that ERDJ3A is a co-chaperone with heat shock proteins (HSPs) expressed in plant cells. The gene could be greatly induced under ER stress in rice and facilitates the delivery of unfolded proteins between vacuoles and ER^25^. We found its expression was elevated 5–8 times in QPM over *o2*, and that a 26,022-bp retrotransposon was located downstream of *ERDJ3A* DNA sequences (Supplementary Fig. 19), which might regulate gene expression due to its large size and spatial change. Whether the insertion affects gene expression and in turn plays a role in endosperm modification remains to be further investigated.

## Discussion

Contiguous sequence information achieved by shotgun DNA sequencing and sequencing technologies advances the molecular genetic analysis of complex genomes in plants and animals. The B73 maize genome was improved with a 52-fold increase in contig length and a notable retrieval of intergenic spaces and centromeres by using single-molecule technologies^10^. Long-read sequences not only uncover extensive SNP and InDel variations, but also identify extensive structural variations between maize inbred lines, which is not apparent from genomes assembled with short reads. The tropical SK maize genome generated from long reads presents 80,614 polymorphic structural variants, across 521 diverse lines and serves as a reference genome to mine genetic variation for crop improvement^9^. Extended contiguous chromosomal information also provides an approach to reconstruct breeding histories. The high-quality Mo17 genome sequence was found to contain extensive intraspecific genome diversity compared to other maize lines, which provided the opportunity to explore the molecular basis of heterosis and genome evolution^11^. In particular, traits composed of multiple QTLs with different weights can be assigned to precise chromosomal regions.

Analysis of maize genome intraspecific variation has important implications for crop improvement and plant breeding. Sequencing an entire genome allows us to reveal the degree of copy number variation and presence/absence variation in maize, which is frequently associated with a change in gene expression and contributes to extraordinary phenotypic diversity^19^. In this study, we identified a high level of gene duplications in the K0326Y genome, indicating potential impact on phenotypic diversity and underlying mechanism of heterosis. There are more than 40 gene copies accounting for the abundant α-zeins that comprise the majority of seed storage proteins in maize^26,27^. The tandem duplications of these genes may be important for breeding, as the expansion and contraction of gene copies could be tied to important quantitative traits. It was shown that a gene duplication conferred enhanced transcript and protein levels of the *γ27* gene, which was proved important for endosperm modification in QPM^8^. The availability of the whole genome allowed us to investigate this and other loci and to make comparative analyses between QPM and non-QPM genotypes. It is possible that knowledge of genomic variation in maize will provide source material to generate alleles. There is evidence that strong artificial selection on specific anthocyanin coloration patterns has often led to the formation of complex alleles with tandem duplications^28^.

Previous molecular characterization of K0326Y QPM was limited by the absence of a complete genome sequence. The *qγ27* QTL was associated with a major *Mo2* based on linkage mapping^15^. To investigate the structure of *qγ27*, a Mo17 BAC clone containing the duplicated *γ27* gene was sequenced^8^, as it would have been extraordinary labor-intensive and time-consuming to determine the structure and expression of any *Mo2* candidate gene in K0326Y without the genome sequence. In this study, we produced a high-quality QPM genome sequence with a contig N50 of 6.99 Mb and captured many sequence polymorphisms and structural variations. We can now trace different introgressions achieved through intensive breeding and thereby uncover multiple potential *Mo2* molecular mechanisms for the development of hard kernel varieties, which can accelerate future breeding via marker-assisted selection.

Holding et al. mapped three major *Mo2* QTLs in K0326Y on chromosomes 1, 7, and 9, using two independently developed QPM lines and recombinant inbred lines (RILs)^3,4^. A microarray hybridization identified 16 differentially upregulated genes in QPM, including *Pfpα* and *γ27*, some of which showed a consistent expression pattern with an increase in vitreous endosperm compared with opaque RILs^3,4^. The PFP protein complex, consisting of two regulatory *α*-subunits and two catalytic β-subunits, is a non-ATP-dependent enzyme that could catalyze a rate-limiting step in glycolysis. It was proposed that increased PFP*α* activity in QPM could attenuate oxygen stress in the endosperm by increasing diminished ATP reserves; PFPβ expression was totally uniform between genotypes^20^. The O2 transcription factor could positively regulated pyruvate orthophosphate dikinase 1 (PPDK1)^8^, as this enzyme is downregulated in *o2* mutants, making it important during late-stage kernel filling. However, an exclusively cytosolic version of PPDK2 that might enhance non-ATP-dependent glycolytic flux was shown to be upregulated in QPM endosperm^20^. We identified another ATP-independent enzyme, cytosolic enolase (ENO), with increased gene expression in QPM. ENO is an integral enzyme in glycolysis, catalyzing the interconversion of 2-phosphoglycerate to PEP. It was reported that enolase transcript levels and enzyme activity were induced by anaerobic stress in maize^29^. This is consistent with a hypothesis that the increased synthesis of non-ATP-requiring enzymes for glycolysis in QPM provides an energy-generating mechanism repressed in *o2*. This mechanism mollifies an energy deficiency, consistent with the previous assumption^20^. RILs generated after seven generations of self-pollination from a cross between K0326Y and W64A*o2* (ref. ^16)^, together with the K0326Y genome sequence, will be valuable to evaluate the contribution of individual *Mo2*s to QPM phenotype.

HSPs, a group of chaperones that assist in protein folding and assembly and maintain optimal conditions for plant growth and development^30^. Our RNA-seq experiments show that most *Hsp* genes are expressed at a higher level in QPM compared to *o2*, indicating that the *o2* mutant may not effectively activate the heat shock response to sustain protein folding. Still, some downregulated *Hsps* in QPM might have other roles due to functional divergence after maize tetraploidization. The effect on HSPs that we detected is reasonably consistent with earlier gene expression data that identified 8 *Hsps* among 37 downregulated genes^20^, compared with 49 (43 increased and 6 decreased) *Hsp* genes in 1791 DGEs in this study. Our increased resolution of gene expression might result from the longer 125-bp paired-end reads^31^, increasing the ability to distinguish homologous Hsp members in two sets of maize sub-genomes, whereas previous study had limited the power to define DGEs by using the shorter 51-bp single-end reads and lower sequence output generated from the early Illumina platform. The 43 of 49 increased HSPs indicates stronger UPRs in QPM. In rice, ERdj3A, which is located in protein storage vacuoles and induced under ER stress, is involved in protein folding and plant immunity^25^. An elevated *ERDJ3A* in QPM could reflect physiological stress created by refolding proteins or facilitating ER-associated protein degradation.

**Fig. 5:**
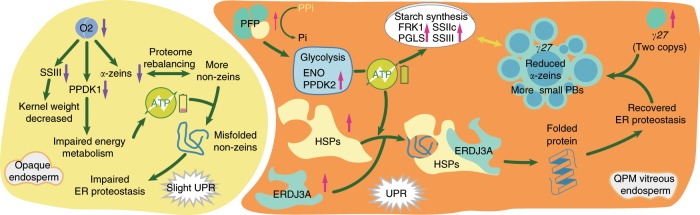
A model illustrating QPM kernel development. The elevated gene expression is marked with a pink arrow, whereas the gene with decreased expression is labeled with a purple arrow. Solid lines represent direct or indirect connections. Not all details of pathway are shown in this diagram in order to simplify the model; more details are in the text. SS starch synthesis, PPDK pyruvate phosphate dikinase, ENO cytosolic enolase, FRK fructokinase, PGLS 6-phosphogluconolactonase, UPR unfolded protein response, ER endoplasmic reticulum, PFP phosphoenolpyruvate, HSPs heat shock proteins, ERDJ3A DnaJ protein.

Based on the previous studies of Holding et al.^20^ and our findings, we propose a model for how starchy endosperm in *o2* is converted into a vitreous phenotype in QPM (Fig. 5). During endosperm development, synthesis of zein proteins are stored in ER PBs, and their synthesis is influenced in a spatio-temporal pattern by ATP availability, which affects their numbers, sizes, and shapes. The inner maize endosperm is deficient in oxygen, and the glycolytic pathway, rather than the tricarboxylic acid (TCA) cycle and oxidative phosphorylation, becomes increasingly important to generate ATP (Supplementary Fig. 20). When normal zein PBs, functional ER, and normal starch biosynthesis occur simultaneously in the presence of sufficient ATP, it facilitates development of a vitreous endosperm^32^. However, the *o2* mutant significantly reduces the amount of zeins and appears to impair normal protein folding in the ER. It was hypothesized that the activation of the unfolded protein response (UPR) in *o2* requires increased amounts of energy in form of ATP and increased levels of chaperons, including heat shock proteins^20^. Moreover, the *o2* mutation has reduced expression of PPDK1, affecting glycolysis (Supplementary Fig. 20) and aggravating the energy shortage^31^. The *o2* mutation also reduces starch synthesis directly, resulting from transcriptional downregulation of genes involved in its synthesis^8^. These metabolic changes impact the way in which PBs and starch grains congeal during kernel desiccation, leading to opacity in the mature endosperm^32^. Alternatively, the pleiotropic effects of *o2* mutations could be explained by additive effects from other processes. The *o2* effect on PB structure through zein gene expression and ATP depletion, through *Pfpα* and *Ppdk1*, could be independent, but they combine to give a more severe endosperm phenotype than reducing zein proteins alone. The recovery of starch biosynthesis and the glycolysis pathway is supported by the increased expression of starch synthetic and glycolysis-related genes in QPM compared to *o2* maize (Supplementary Fig. 21). This is consistent with multiple *Mo2*s involved in different metabolic pathways required to restore *o2* mutants to a vitreous phenotype (Fig. 5).

## Methods

### Plant material

The maize (*Zea mays*) inbred line K0326Y was selected for sequencing because of its previous role in the study of QPM. The seeds were obtained from the Holding laboratory at the University of Nebraska, Lincoln. For DNA extraction, plants were grown in a greenhouse at 25 °C for 12 days, then treated in darkness for 3 days to minimize chloroplast DNA; leaves of seedlings were harvested and frozen immediately in liquid nitrogen for extraction of DNA. Genomic DNA for library construction was obtained using the PacBio protocol^33^. The quality of the DNA was assessed with Qubit, NanoDrop 2000, and pulsed-field electrophoresis.

To compare varying degrees of endosperm modification for *o2* mutants, 100-kernels of a QPM population, O2POOL 42, segregating *Mo2*s were obtained from the International Maize and Wheat Improvement Center, CIMMYT (http://mgb.cimmyt.org/gringlobal/accessiondetail.aspx?id = 14778, plant ID 014785). They were grown in Sanya, Hainan.

### PacBio and Illumina library construction and sequencing

Libraries for SMRT PacBio genome sequencing were constructed by using high molecular weight and high-quality DNA^33^. More than 10 μg of high-quality genomic DNA was sheared to ~20 -kb using g­TUBE (Covaris). Sheared genomic DNA was treated for damage repair and end repair, adaptor ligation, and size selection with a Blue Pippin system (Sage Science) to create SMRTbell template libraries. The final libraries were sequenced on the PacBio Sequel platform (Pacific Biosciences, CA, USA) using Sequel Sequencing Kit 2.1 Bundle. Libraries for Illumina PCR-free paired-end genome sequencing were constructed according to the manufacturer’s standard protocol (Illumina, CA, USA). Approximately 5 μg of genomic DNA was fragmented, then size-selected (350 bp) by agarose gel electrophoresis. The ends of selected DNA fragments were blunted with an A-base overhang and ligated to sequencing adapters. After quality control, all the PCR-free libraries were sequenced on an Illumina platform with a paired-end sequencing strategy.

### De novo assembly of PacBio SMRT reads

A total of 28 million PacBio post-filtered reads with an N50 read length of 16 kb was generated from 93 SMRT cells. A read length above 8 kb was used for contig assembly with Falcon^34^. The Illumina paired-end reads were mapped to contigs with BWA-MEM^35^ and used to polish the assembly with Pilon^36^.

### Construction of BioNano optical maps

The assembled genome was used to identify restriction sites for BioNano optical maps sequencing with LabelDensityCalculator 1.3.0. BspQ1, a restriction endonuclease that recognizes the target site of GCTCTTC was specifically selected due to its proper label density distributed in the K0326Y genome. The samples were loaded into chips and then the molecules were collected with the BioNano Irys system. The raw BNX files were filtered and assembled into genome maps by using BioNano Solve pipeline (see URLs).

### Hybrid assembly of PacBio contigs and BioNano optical maps

To make hybrid scaffolds, the previous contigs and the optical maps were aligned using the module ‘HybridScaffold’ in the BioNano Solve pipeline, with the merge P-value of 1 × 10^−11^ and alignment length of 80 kb. The gaps were filled by using corrected PacBio reads with PBjelly of the parameters ‘< blasr > -minMatch 8 -minPctIdentity 75 -bestn 1 -nCandidates 20 -maxScore −500 -nproc 4 –noSplitSubreads < /blasr > ’.

### Construction of pseudomolecules

The reference genome of B73 (ref. ^10)^ was used to anchor K0326Y scaffolds to chromosomes. Contigs were ordered using NUCmer^37^ and placed on chromosomes based on the synteny of K0326Y and B73.

### Assembly evaluation

The maize GBS-tag sequences (~4.4 million) were mapped against the K0326Y genome with BWA-MEM^35^ to evaluate the assembly. The Illumina paired-end data were also used to evaluate the assembly accuracy with the mapping program of SOAP2 (ref. ^38)^. Genome completeness was assessed by a number of 1440 single-copy orthologous genes in the Embryophyta dataset of BUSCO^13^.

### Repetitive element annotation

The transposable elements were analyzed with a combination of homolog-based and de novo approaches. The K0326Y-specific TE library was built by RepeatModeler^39^. The final repetitive elements were defined with RepeatMasker^40^ by using K0326Y-specific and Repbase library^41^. The full long terminal repeats (LTR) retrotransposons were predicted using LTRharvest^42^ and annotated by LTRdigest^43^. *Helitrons* were predicted by the HelitronScanner program^44^. SINE-finder^45^ was applied to search for tRNA-derived SINEs in the forward strand of the entire genome. Long interspersed nuclear elements (LINEs) were identified using TARGeT^46^. Terminal inverted repeats (TIRs) were predicted via the pipeline of TARGeT^46^. MITEs were annotated by MiteHumter^47^ based on the Matlab toolbox.

### Evolutionary dynamics of retrotransposition

A unique identifier including the information of superfamily and family was assigned to each structurally defined LTR in the genome. Families were predicted for each TE superfamily using the 80–80–80 rule by implementation in SiLix^48^ and Vsearch^49^. The 5’-end sequences of LTR retrotransposons were used to cluster families^10^. To investigate the evolutionary dynamics of retrotransposition in maize, the consensus sequences from LTR families with 80% divergence were extracted and aligned with MAFFT^50^. The maximum likelihood phylogenetic trees were constructed by RAxML^51^ and visualized via iTOL (https://itol.embl.de/)^52^.

### Identification of centromeric regions

The centromere-related long terminal repeat (CRM), retrotransposons, and the 156-bp tandem repeats (CentC)^14^ were used to identify the centromeric regions. The CRM sequence was aligned with the K0326Y genomes by BLASTN, with sequence similarity ≥80% and e-value ≤1e–20. After filtering, we used the R script to calculate the candidate centromeric region for chromosomes with 95% confidence interval. The CentC was identified using tandem repeat finder (TRF, Version 4.07b) with the parameters of ‘1 1 2 80 5 200 2000 -d’. More than 10 copies of CentC were defined as the candidate centromere region. Overlapping regions from the two methods were used to determine the centromeric regions.

### Iso-seq and RNA-seq

To optimize the collection of gene expression data, RNAs from nine tissues (Roots, stems, 12-d seedlings, silks, tassels, ears, whole seeds, callus, and young leaves) were sampled using TRIzol reagent (Invitrogen, Carlsbad, CA) and pooled in equal amounts. Enriched polyA RNA was reversely transcribed into cDNA by using the Clontech SMARTER cDNA synthesis kit, which was subjected to size selection (1~2 kb, 2~3 kb, 3~6 kb, 5~10 kb) using BluePippin. The pooled libraries were sequenced in 40 SMRT cells on the RSII platform and 12 cells on the Sequel platform. PacBio raw data were processed following SMRT analysis using the Iso-seq pipeline to generate Circular Consensus Sequences (CCS). The full-length non-chimeric (FLNC) transcripts were classified and polished using the Iterative Clustering and Error Correction (ICE) algorithm. The high-quality isoforms were mapped to the genome by GMAP^53^, collapsed with Cupcake ToFU to reduce redundancy. RNA-seq libraries were prepared according the manufacturer’s protocol of Illumina and paired-end sequenced with an insertion size of 350 bp. The reads were assembled using Trinity^54^ and clustered by TIGR^55^.

### Gene annotation

The K0326Y genome was annotated with the pipeline of MAKER-P^15^ based on comprehensive evidence from homologous protein sequences, K0326Y transcripts, and ab initio prediction. We used the same repeat masking library that was used for B73 annotation^10^, with the addition of the LTR library annotated by LTRharvest. The gene models were generated by the programs of Augustus^56^ and FGENESH (see URLs) trained for maize and other monocots. The protein sequences were downloaded from Ensemblgenomes release-41, including the species of *Arabidopsis thaliana*, *Oryza sativa*, *Setaria italica*, *Sorghum bicolor*, and *Zea mays*^57^. K0326Y transcripts were derived from assembled RNA-seq reads and PacBio isoform sequences. The database of Swiss-Prot, TrEMBL, Interpro, nr, KEGG, KOG, and GO were chosen to assist in assigning functional properties to K0326Y gene models using BLASTP^58^.

### Comparative genomics and structural variation identification

Genome alignment among K0326Y, Mo17, and B73 was performed using NUCmer from Mummer package^59^ with the parameters of ‘-mumreference -g 1000 -c 90 -l 40’. The delta-filter was used to remove the mapping noise and determine the one-to-one alignment blocks with parameters ‘-r -q’. SNPs and InDels (<100 bp) were extracted using show-snp in the filtered one-to-one aligned blocks. All these variants were annotated using the ANNOVAR program^32^. Gene duplications were analyzed with BLASTP (e-value < 1e-10, -v 5, -b 5) for the pairwise similarity measurement and MCscanX package for classification^60^. The rates of Ka and Ks were calculated with PAML^61^. Gene duplications were analyzed with BLASTP for the pairwise similarity measurement and MCscanX package for classification^60^. The presence/absence variations (PAVs) that was only present in K0326Y but absent in B73/Mo17 were identified as following. We divided the K0326Y genome into 1000-bp overlapping windows with a step size of 200 bp, which was aligned against B73/Mo17 with BWA-MEM^35^ and options of ‘-w 1000–M’. The fragments that could not be aligned or alignment coverage was less than 25% were assigned as K0326Y-specific sequences. Then we aligned the CDS of longest transcript to K0326Y-specific sequences by GMAP, and defined CDS regions that covered by K0326Y-specific sequences over 75% as PAV genes. For structural variations, we aligned corrected PacBio long reads from K0326Y against B73 and Mo17 with NGMLR^62^ using default parameters. Structural variations (SVs) were called using Sniffles^62^ when more than 10 reads were supported. Sequences of SVs were retrieved based on their location using BEDTools^63^, identified with the ANNOVAR program^43^.

### Bulked-segregant analysis

K0326Y (QPM) and W64A*o2* (a classic *o2* mutant) were used to construct an F_2_ population. The extreme vitreous and opaque seeds of F_2_ generation were planted in greenhouse. Their DNA samples were screened using the 0707-1 primer^8^ and only the ones containing the *qr27* allele were pooled, amounting to 165 plants with extreme vitreous phenotype and 160 seedlings with extreme opaque phenotype. The DNA library was sequenced with 2 × 150 read lengths and 80-fold depth using the Illumina platform. The raw reads were mapped using BWA-MEM^35^ and SNPs were called using GATK^64^. A statistical method through calculating the SNP-index and G’ values were applied for detecting the closely linked SNPs for the target traits in each bulk^26,65^. SNP-index and G’ values were calculated in 4 Mb windows with a sliding of 10 kb. To analyze the linkage between the *Helitron* insertion into the promoter of *Pfpα* and the vitreous endosperm kernels of the segregating F_2_ population, a PCR reaction was performed using a pair of gene specific primers (F: TCTATTTTGCGGTTCTA; R: CTCCCAGCCTAAGCCTC).

### Transcriptome differential expression analysis

The maize inbred lines of K0326Y, W64A*o2*, CM105*Mo2*, and CM105*o2* were grown in a field in Shanghai, China. Total RNA from endosperm collected at 16 DAP was extracted using TRIzol reagent (Invitrogen). RNA-seq libraries were prepared according to the manufacturer’s protocol for Illumina and sequenced to generate 125-bp paired-end reads using the Illumina HiSeq platform. Adapters and Low-quality reads were removed using Trimmomatic^66^. The clean reads were aligned to the maize K0326Y genome using Hisat2 (ref. ^67^) with the built-in Bowtie2 mapping program. Unique mapped reads were counted with htseq-count (HTSeq)^68^. The DEseq2 (ref. ^69)^ package based on the negative binomial distribution was used to normalize the RNA-seq data and to identify differential expression genes with the standard of the adjusted P-value of 0.05 (Likelihood Ratio Test, sample size *n* = 3) and the fold-change of more than 1.5×. The PFPα gene expression was also quantified and validated by qRT-PCR reaction by using the specific primers (F: TACTACTTTATTCGGATGATGG; R: CAGAAACCTCCTCGCCTA). The maize *Actin* gene was used as a reference control (F: GCTACGAGATGCCTGATGGTC, R: CCCCCACTGAGGACAACG).

### URLs

Arrow, https://github.com/PacificBiosciences/GenomicConsensus/; BioNano Solve pipeline, https://bionanogenomics.com/support-page/bionano-solve/; FastQC, www.bioinformatics.babraham.ac.uk/projects/fastqc/; FGENESH, http://www.softberry.com/berry.phtml/; ToFU, https://github.com/Magdoll/cDNA_Cupcake.

### Reporting summary

Further information on research design is available in the Nature Research Reporting Summary linked to this article.

## Supplementary information

Supplementary Information

Peer Review

Reporting Summary

Description of Additional Supplementary Files

Supplementary Dataset 1

Supplementary Dataset 2

Supplementary Dataset 3

Supplementary Dataset 4

## Data Availability

Data supporting the findings of this work are available within the paper and its Supplementary Information files. A reporting summary for this Article is available as a Supplementary Information file. The datasets generated and analyzed during the current study are available from the corresponding author upon request. All datasets reported in this study have been deposited in GenBank (NCBI) with the following accession IDs: Genome assembly, VAUS00000000 [https://www.ncbi.nlm.nih.gov/nuccore/CM018593.1]; Raw data for genome assembly and annotation, PRJNA539996; Raw data for Bulked-Segregant Analysis, PRJNA578235; and RNA-seq of QPM and o2 maize, PRJNA578012. The source data underlying Figs. 2, 4b, as well as Supplementary Figs. 2b, 5, 9, 10, 13b, 18b, 19b, and 21 are provided as a Source Data file.

## Source data

Source Data
