# Supplementary material for: Long-read sequencing reveals genomic structural variations that underlie creation of quality protein maize
Source: Nat Commun. 2020 Jan 7;11:17. doi: 10.1038/s41467-019-14023-2 (PMC6946643; doi:10.1038/s41467-019-14023-2)
Supplement: Supplementary file 1 — Supplementary Information [file 41467_2019_14023_MOESM1_ESM.pdf]

Long-read sequencing reveals genomic structural variations that underlie creation of Quality Protein Maize

Li *et al.*

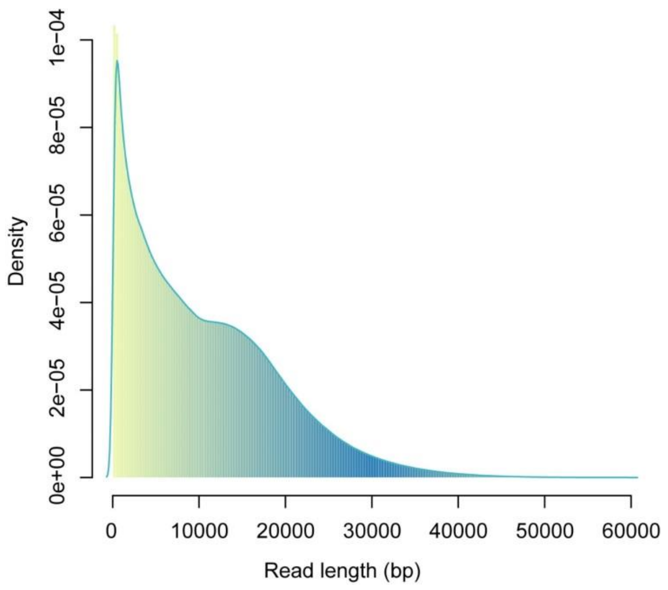

Supplementary Fig. 1. Length distribution of PacBio subreads. The long reads were generated from 93 SMRT cells using PacBio Sequel platform, equal to ~139-fold coverage of K0326Y genome (2.16 Gb) with a N50 subread of 16.6 kb. The x-axis showed the read length and the y-axis was the read density.

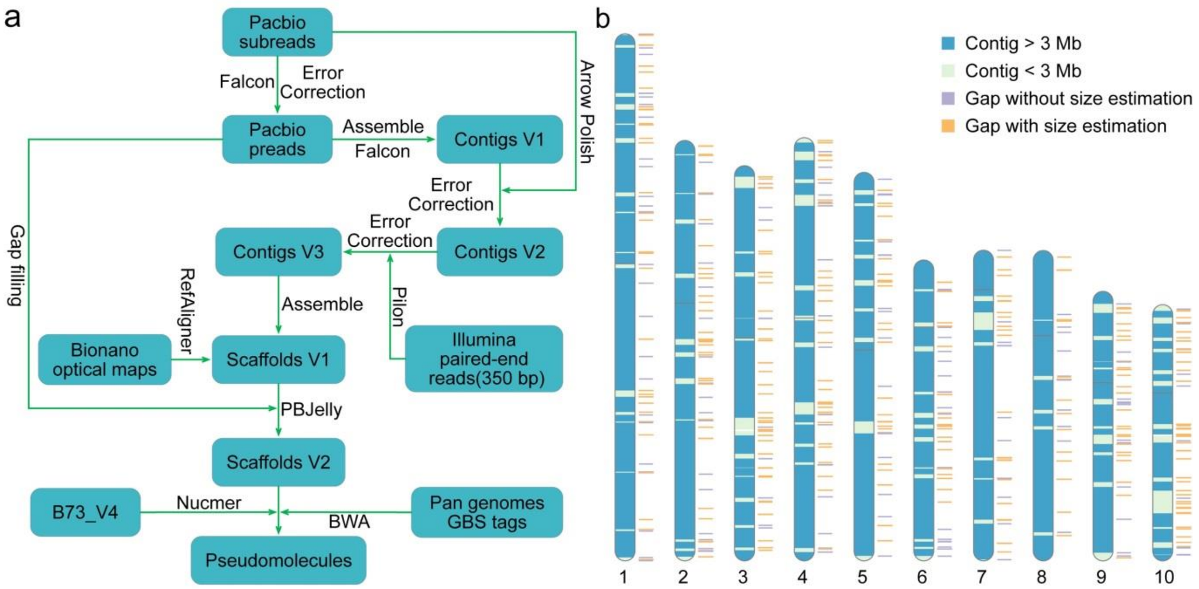

Supplementary Fig. 2. Genome assembly pipeline and chromosome layout. (a) Flow chart of K0326Y genome assembly. The PacBio long reads were assembly by Falcon into 1,221 contigs. After correction, the contigs were integrated into 870 scaffolds with the information of Bionano optical maps. The gaps were filled with PBJelly. Given the synteny of maize genomes, K0326Y pseudomolecules were generated with the integration of B73 and GBS tags. (b) Ideograms of K0326Y pseudomolecules. More than 88% of the assembled genome was constituted of contigs longer than 3 Mb shown as blue bars, while the left was shown as light green in the left track. The right track showed positions of 438 gaps in the pseudomolecules, including 165 gaps with estimated sizes from optical maps (orange) and 273 gaps (purple) without size determination. Source data underlying Supplementary Figure 2b are provided as a Source Data file.

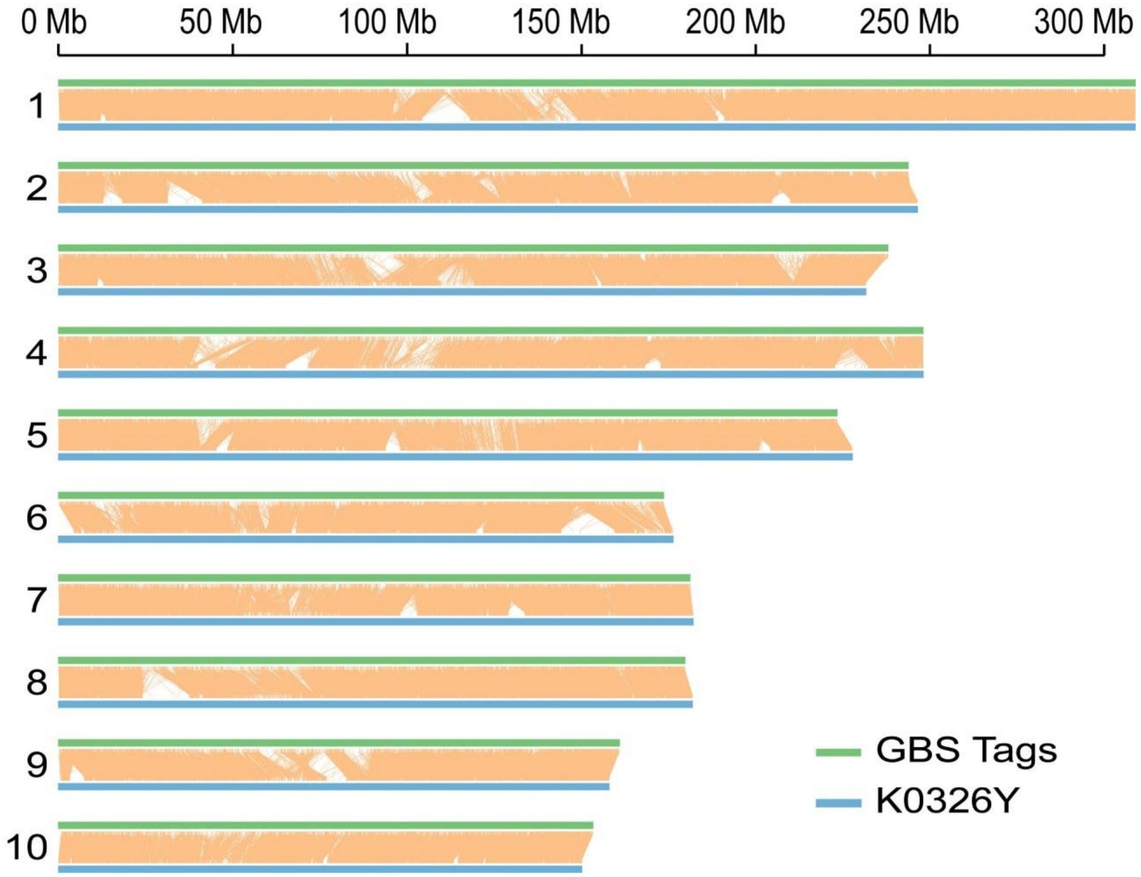

Supplementary Fig. 3. The alignment of GBS tags against the assembled K0326Y genome. The orange lines indicated that GBS tags were aligned to the K0326Y. The top green horizontal line showed GBS tags and the bottom blue line indicated K0326Y genome.

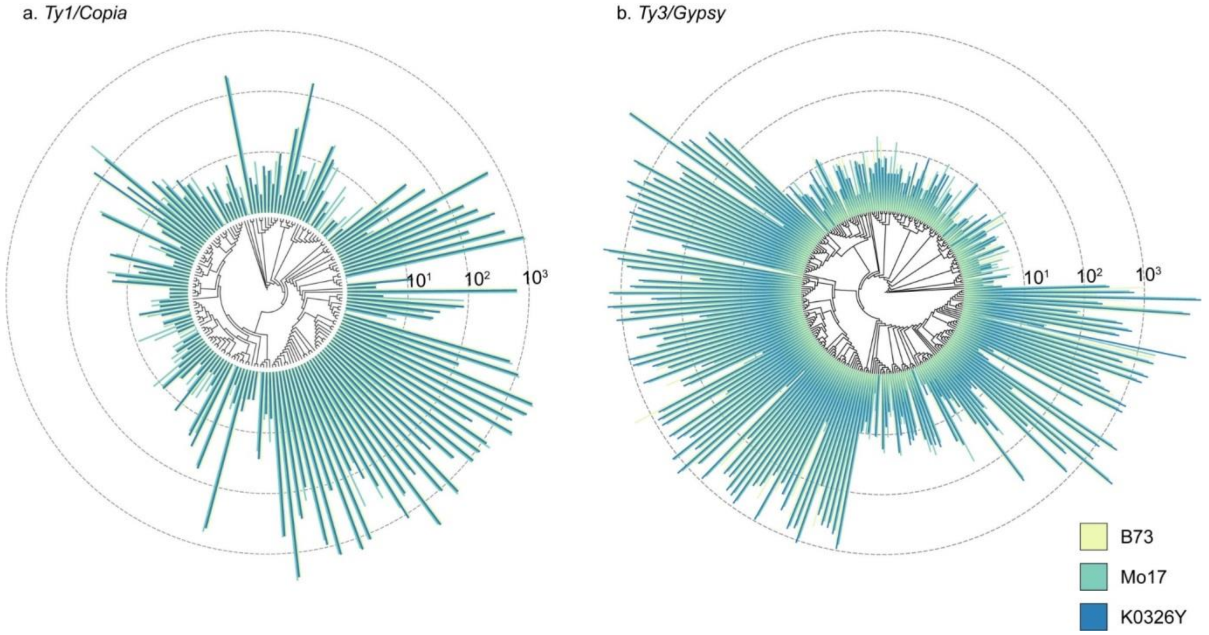

Supplementary Fig. 4. Phylogeny of LTR retrotransposon families in maize inbred lines of K0326Y, B73 and Mo17. (a) *Ty1/Copia*. (b) *Ty3/Gypsy*. The radius length represented copy number of repeats. The yellow, green and blue lines were corresponding to B73, Mo17 and K0326Y, respectively.

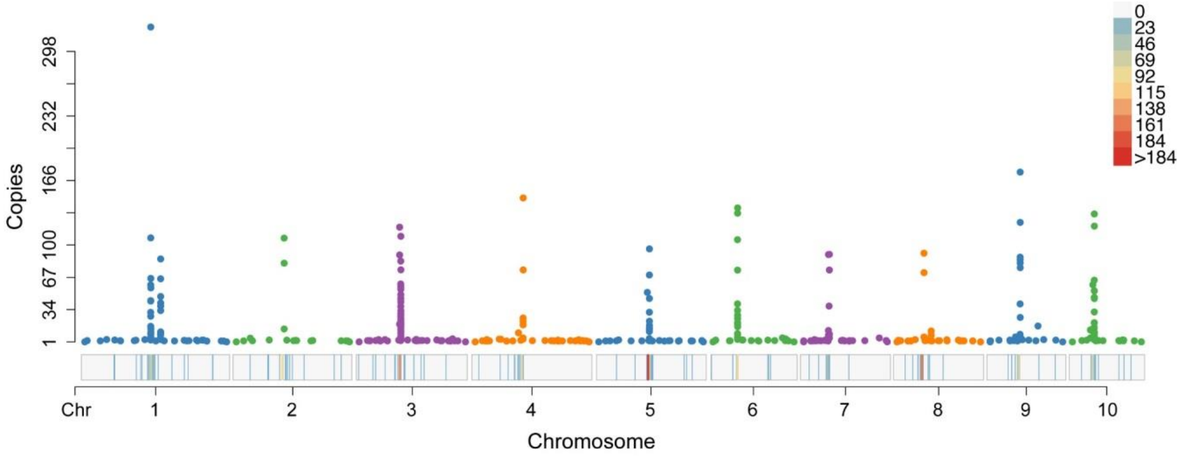

Supplementary Fig. 5. Characterization of centromeres in K0326Y. The Manhattan plot showed the copy numbers of CentC (156-bp tandem repeats) in each chromosome. The heatmap showed the distribution of CRM (centromere-related long terminal repeat) in a 4-Mb window. Source data are provided as a Source Data file.

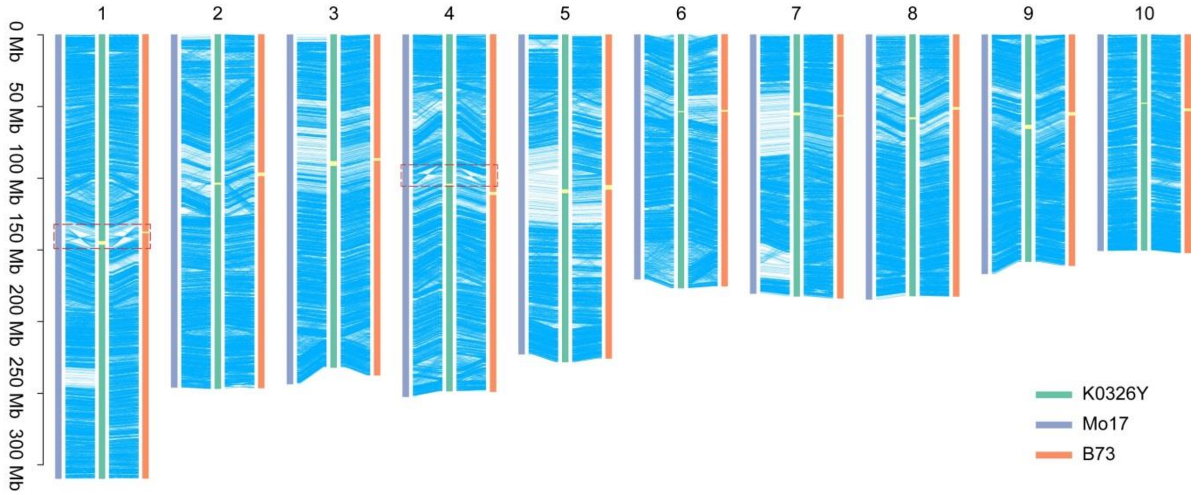

Supplementary Fig. 6. Whole-genome comparison of K0326Y versus B73 and Mo17. Blue lines represented the one-to-one aligned regions between aligned pseudomolecules. Red dashed boxes showed two inversions in chromosome 1 and 4. The fluorescent yellow blocks indicated pericentromere regions in K0326Y and B73 genome. The pericentromere regions of Mo17 were not shown.

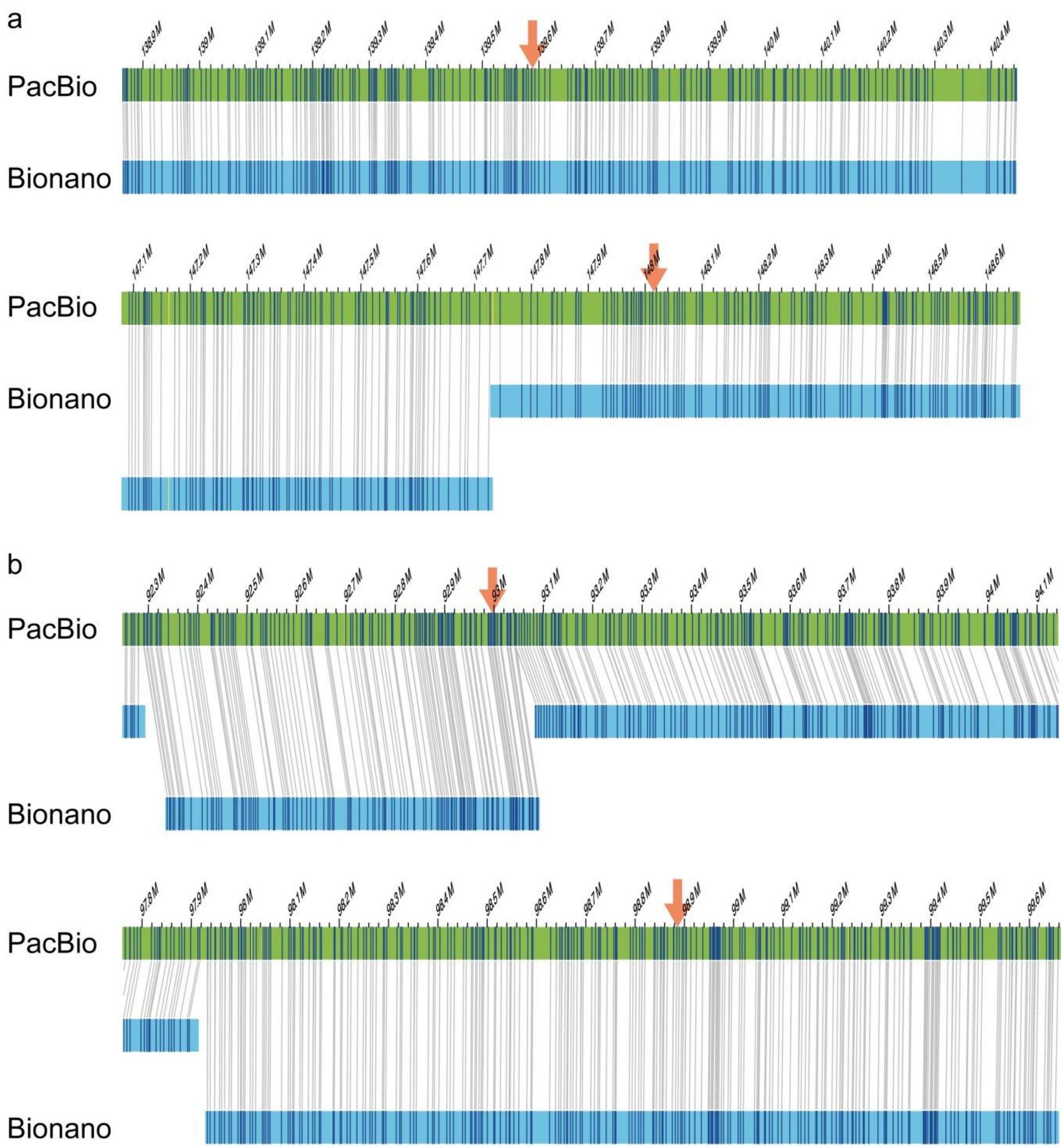

Supplementary Fig. 7. Inversion validations. (a) The inversion in K0326Y chromosome 1 derived from PacBio genome assembly was confirmed by BioNano physical map. (b) The inversion in chromosome 4 from PacBio genome assembly was consistent with BioNano data. The orange arrows indicated breakpoints in B73 genome comparisons. Grey lines showed collinearity between PacBio assembly and enzyme sites in BioNano contigs.

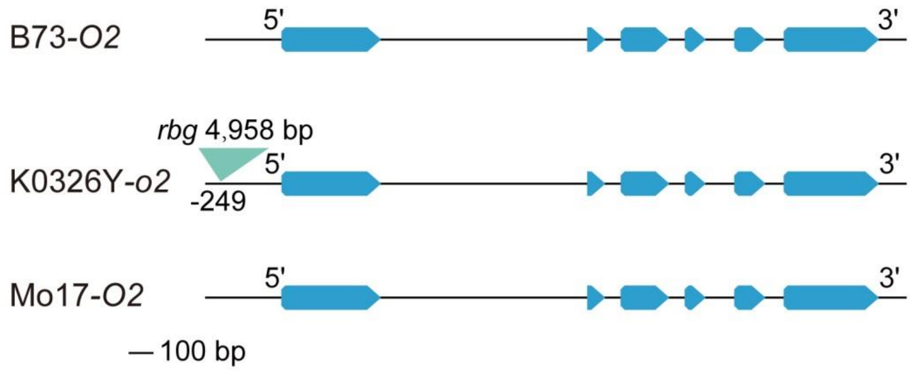

Supplementary Fig. 8. *O2* gene structure. The *o2* gene in K0326Y, B73 and Mo17 were aligned and annotated, showing six exons in blue pentagons. There was a *rbg* transposon insertion with a size of 4958-bp located at 249-bp upstream of the initiator codon (ATG) in K0326Y, labelled as a green triangle.

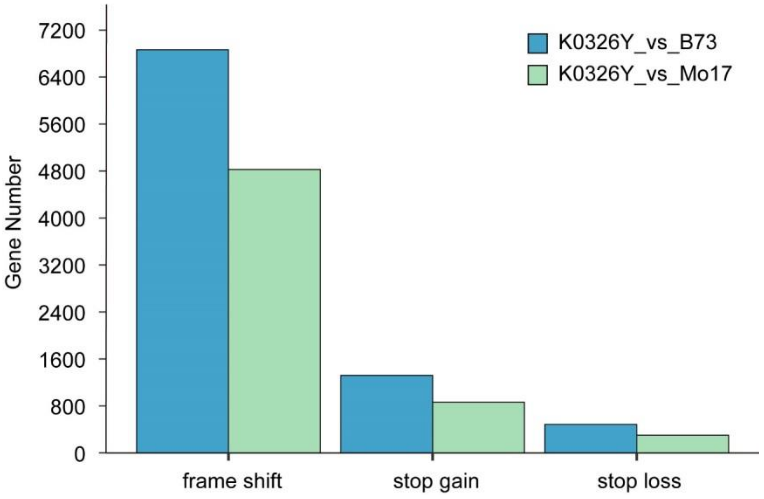

Supplementary Fig. 9. The number of genes affected by SNPs and small InDels. The sequence polymorphism of SNPs and InDels effected gene variations of frameshift, gain of stop codon and loss of stop codon. The blue histogram showed data between K0326Y and B73, and the green histogram showed data between K0326Y and Mo17. Source data are provided as a Source Data file.

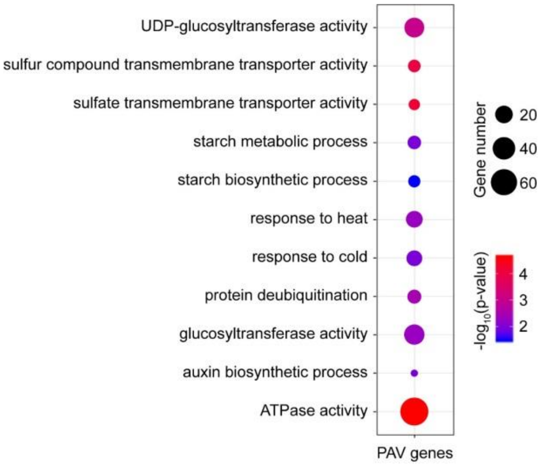

Supplementary Fig. 10. Gene ontology enrichment analysis of up-regulated genes between QPM and *o2* mutant in presence/absence variations. Source data are provided as a Source Data file.

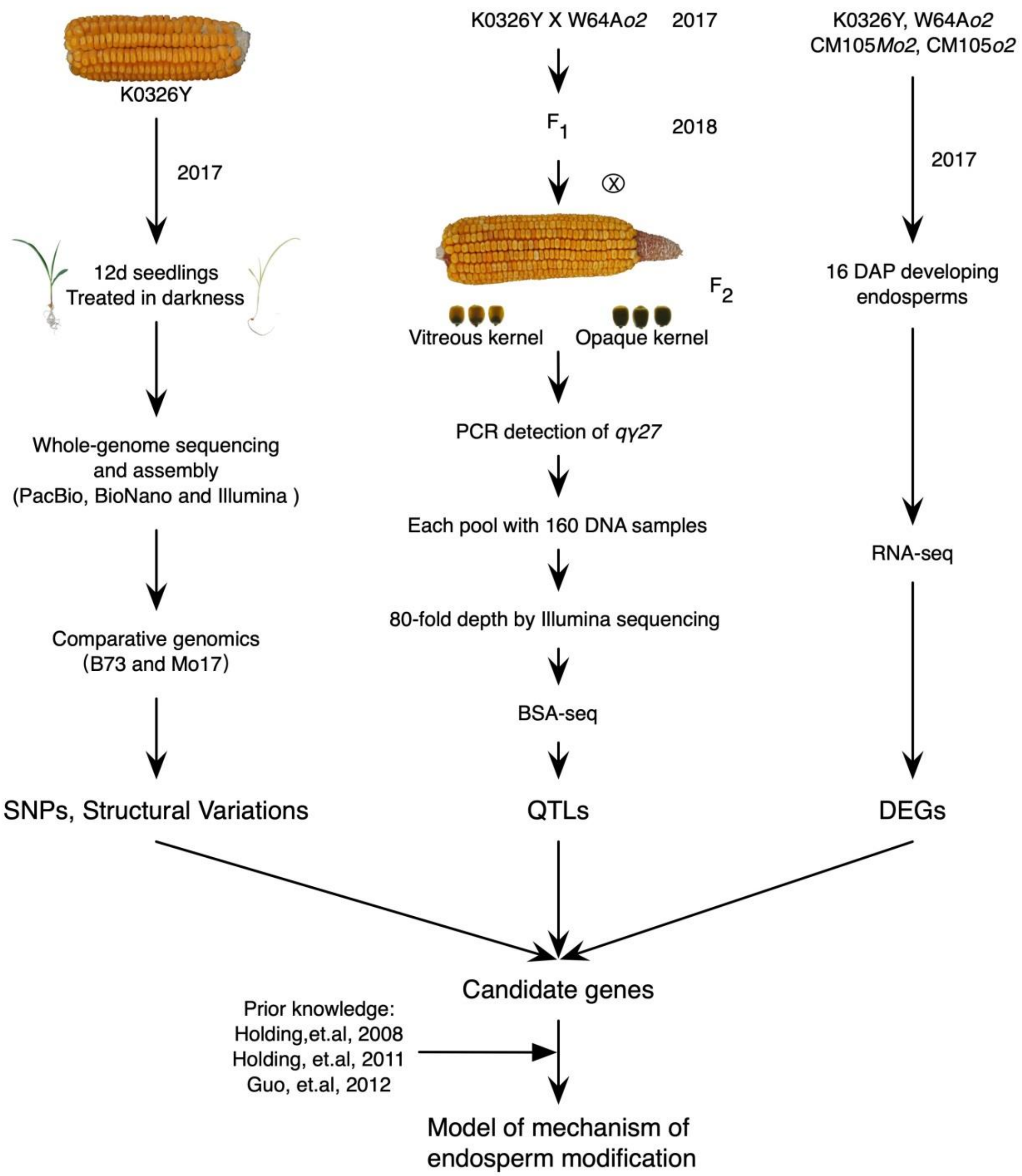

Supplementary Fig. 11. Flow chart of bulk segregant analysis for *o2* modifiers. K0326Y and W64A*o2* was crossed in 2017 and selfed to generate F_2 population in 2018. The opaque and vitreous kernels were selected based on the *o2* modifier1 of *qr27* (two copies of 27-kDa genes). The pooled DNA samples were subjected to BSA-seq. Two sets of QPMs and *o2* mutants （K0326Y and W64Ao2, CM105*Mo2* and CM105*o2*） were used to generate differentially expressed genes (DEGs). Combined with the sequence variations, the QTL regions and DEGs, as well as prior knowledge, we generated candidate *o2* modifiers and proposed a model of mechanism of endosperm modification.

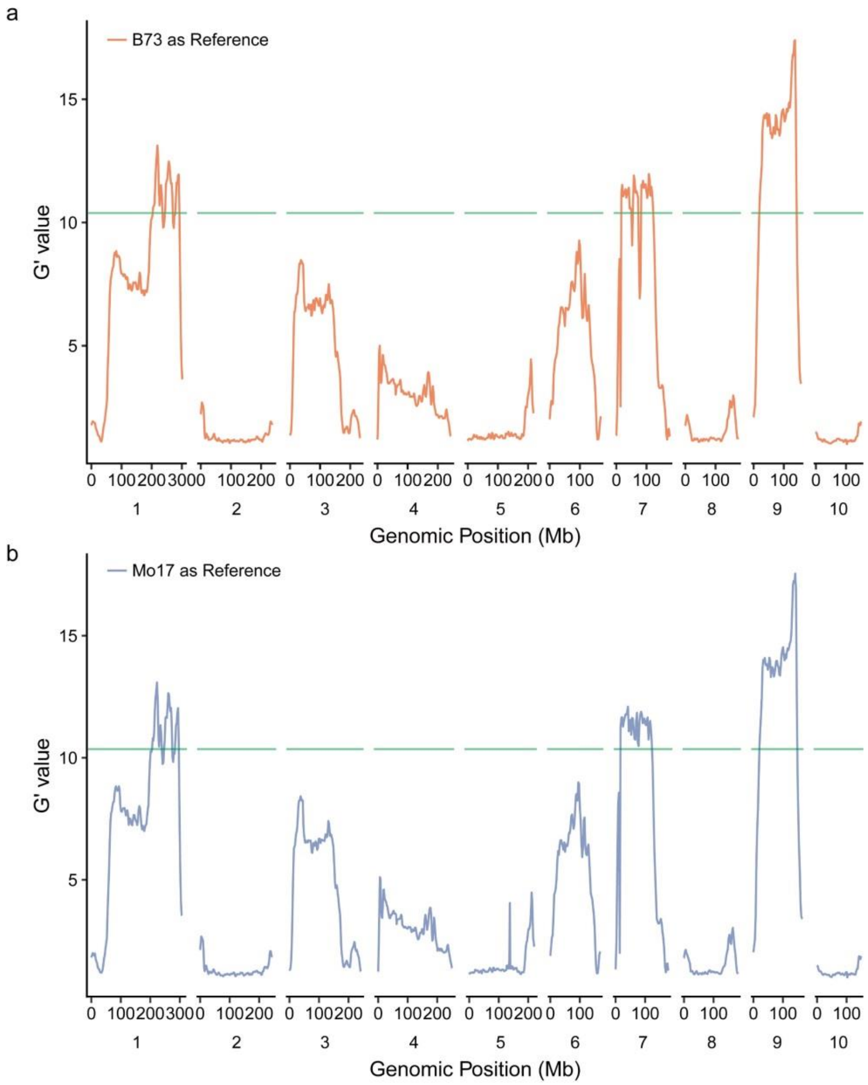

Supplementary Fig. 12. Bulk segregant analysis of a segregating population derived from K0326Y and W64A*o2* cross. (a) Bulk segregant analysis (BSA) using B73 as a reference. (b) Bulk segregant analysis (BSA) using Mo17 as a reference. The G' value is a smoothed version of the standard G statistic in each 4-Mb sliding window. Green line indicates threshold of G' value corresponding to a FDR of 8×10^{-7}.

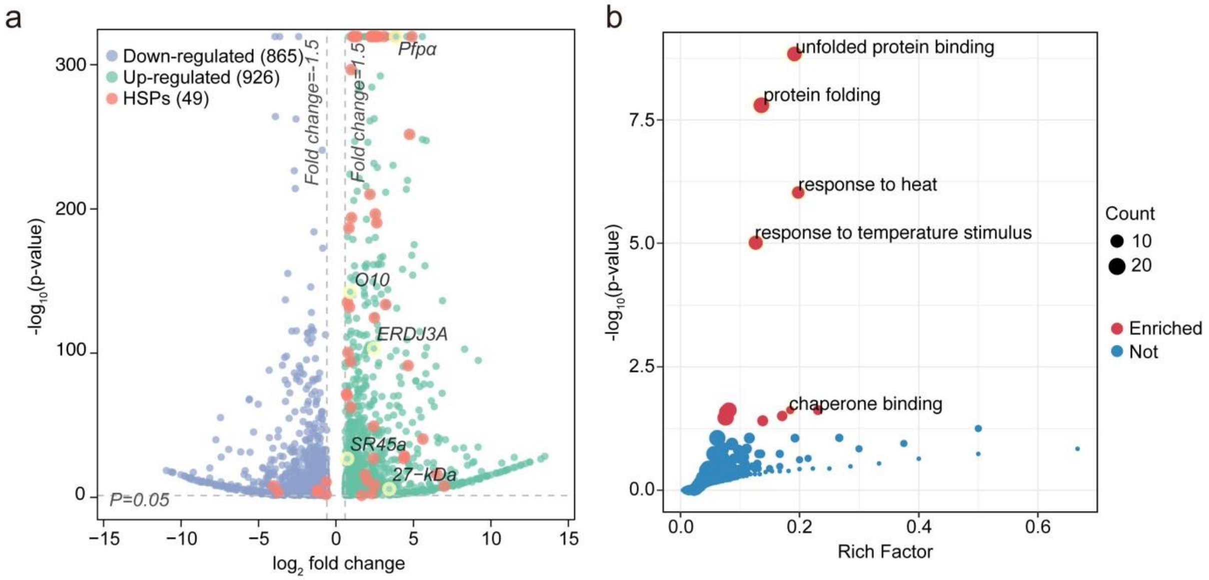

Supplementary Fig. 13. Differential expression of genes and GO enrichment between QPMs and *o2* mutants. (a) Volcano plot of differentially expressed gene between two sets of vitreous QPMs and soft *o2* mutants (K0326Y against W64A*o2*, CM105*Mo2* against CM105*o2*). The up-regulated genes were shown in green and the down-regulated genes were illustrated in light purple. The heat shock related proteins (HSPs) were shown in orange. (b) Gene ontology (GO) enrichment analysis of for up-regulated genes in QPMs. The Rich Factor was the ratio of differentially expressed gene numbers against all annotated gene numbers in same GO term. Source data underlying Supplementary Figure 13b are provided as a Source Data file.

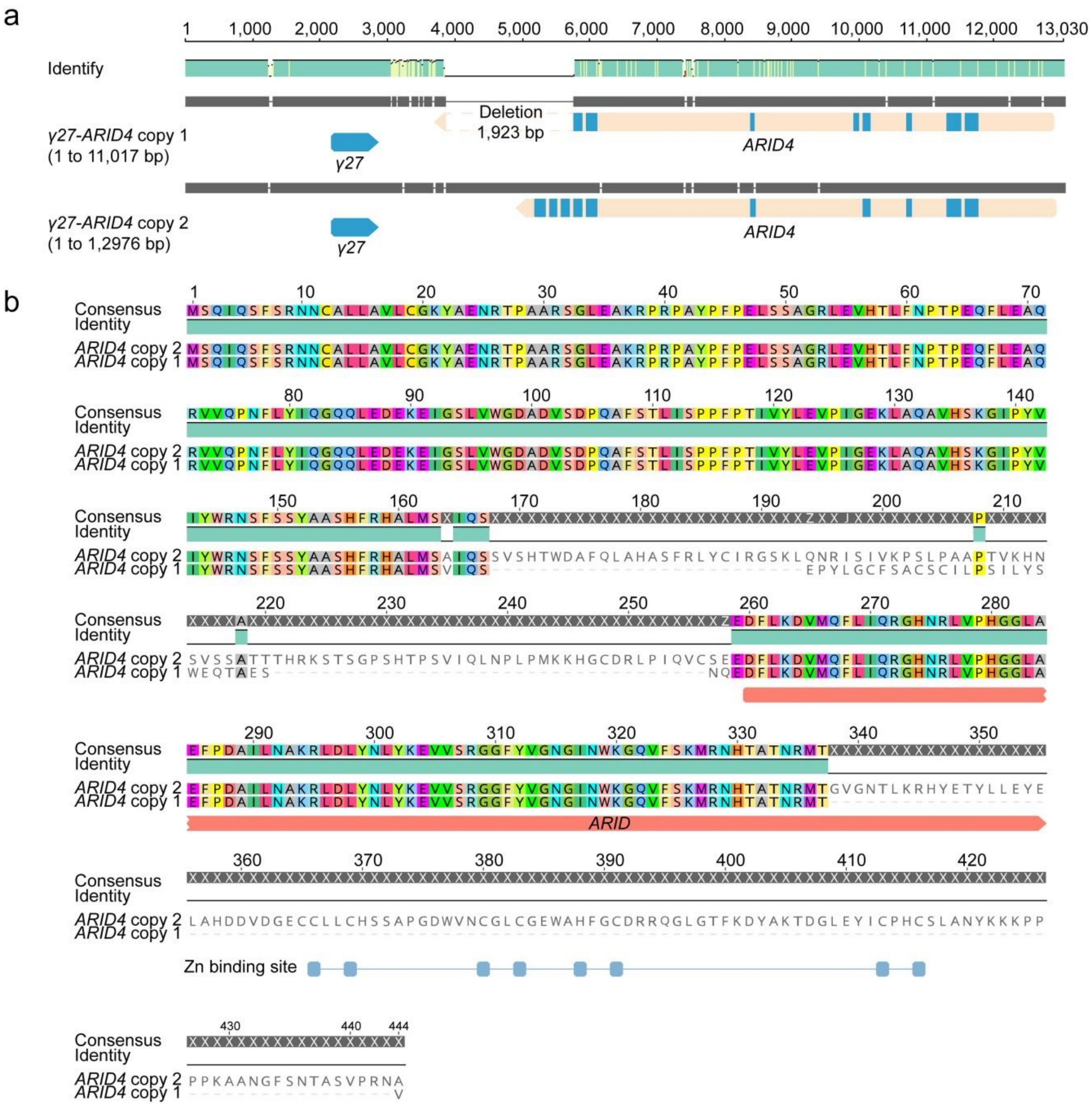

Supplementary Fig. 14. The difference between two copies of *γ27* and ARID-transcription factor 4 (*ARID*). (a) The alignment between two copies of *γ27* and *ARID*. The gene structure was shown with exons in blue and introns in light brown, respectively. The deletion at the 3' end of *ARID* copy 1 was presented in a white box. (b) The alignment of *ARID* protein sequences. The Zn binding site missing in copy 1 was highlighted.

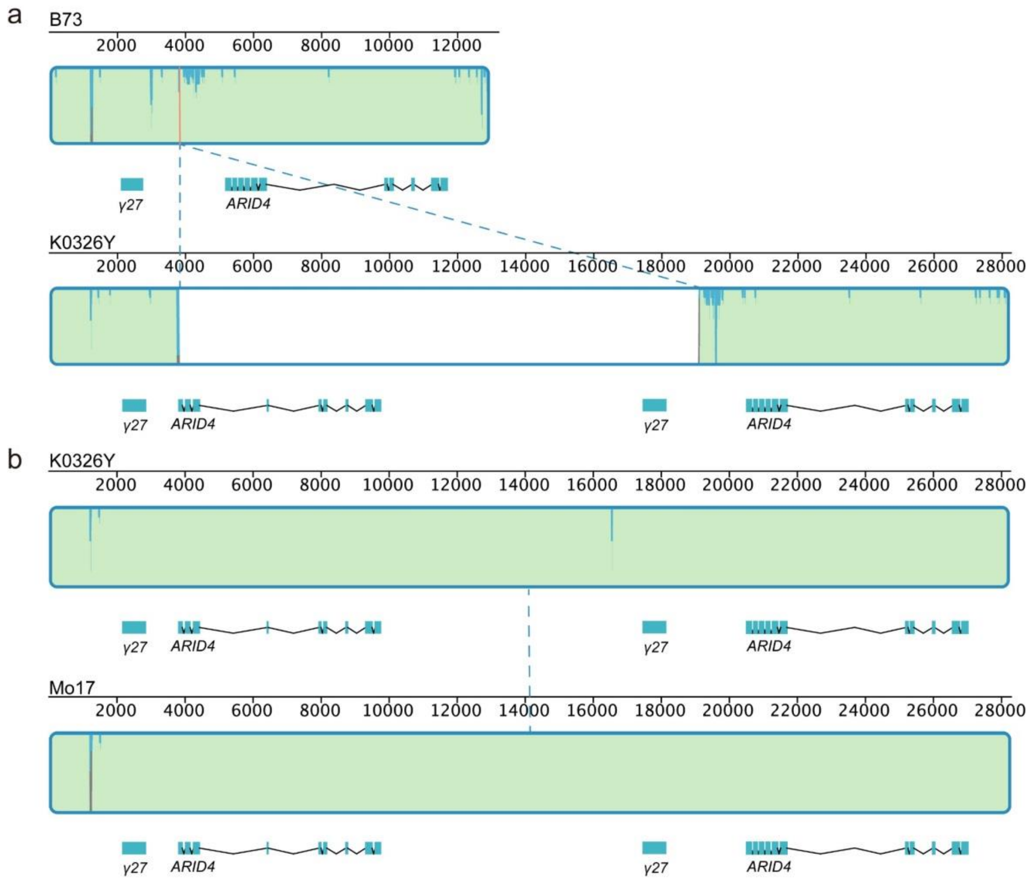

Supplementary Fig. 15. Global alignment of *γ27* loci among B73, K0326Y and Mo17. (a) Global alignment of *γ27* loci between B73 and K0326Y. (b) Global alignment of *γ27* loci between K0326Y and Mo17. The upper and lower tracks represent the alignment identify and gene structure, respectively.

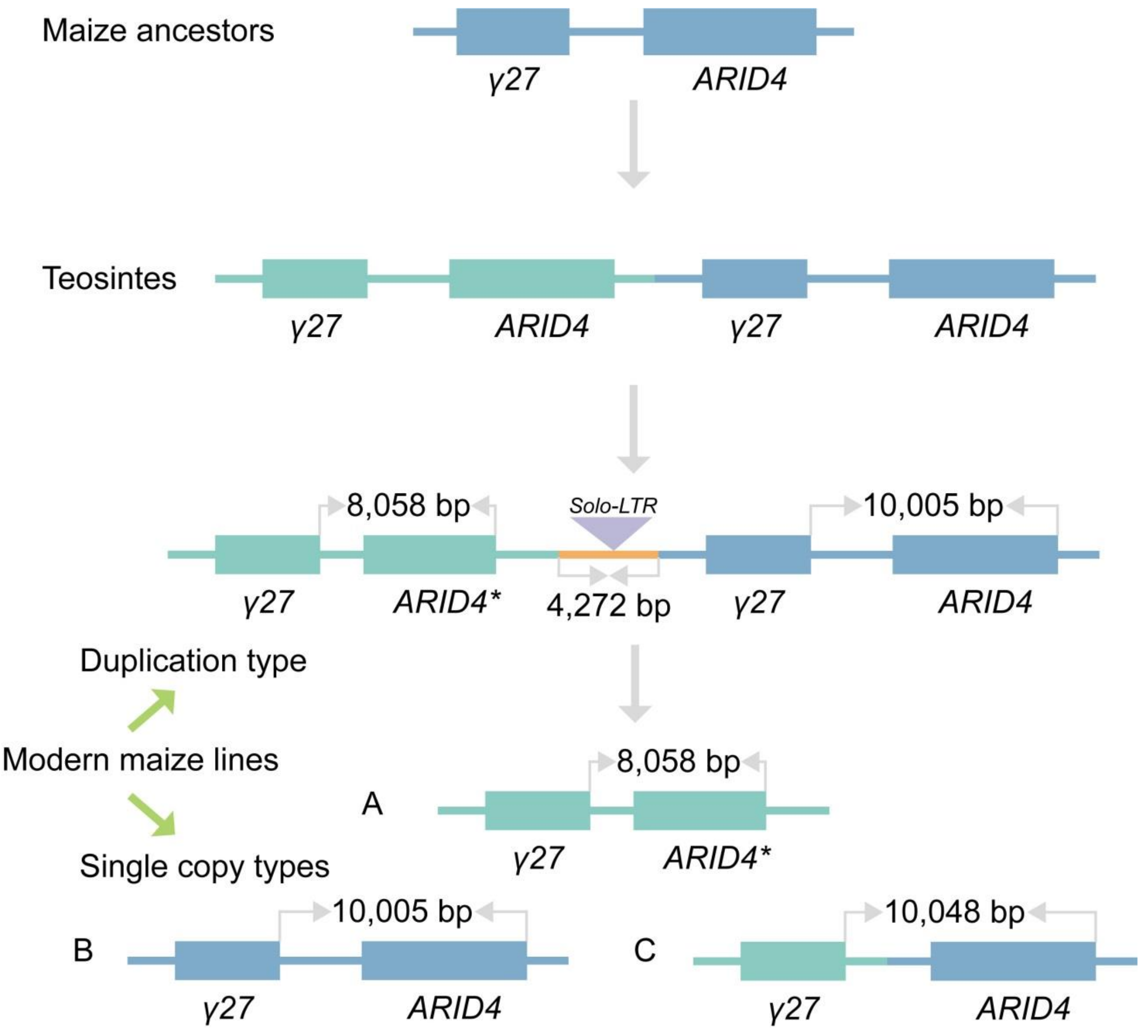

Supplementary Fig. 16. Evolution and natural selection of *γ27* loci.

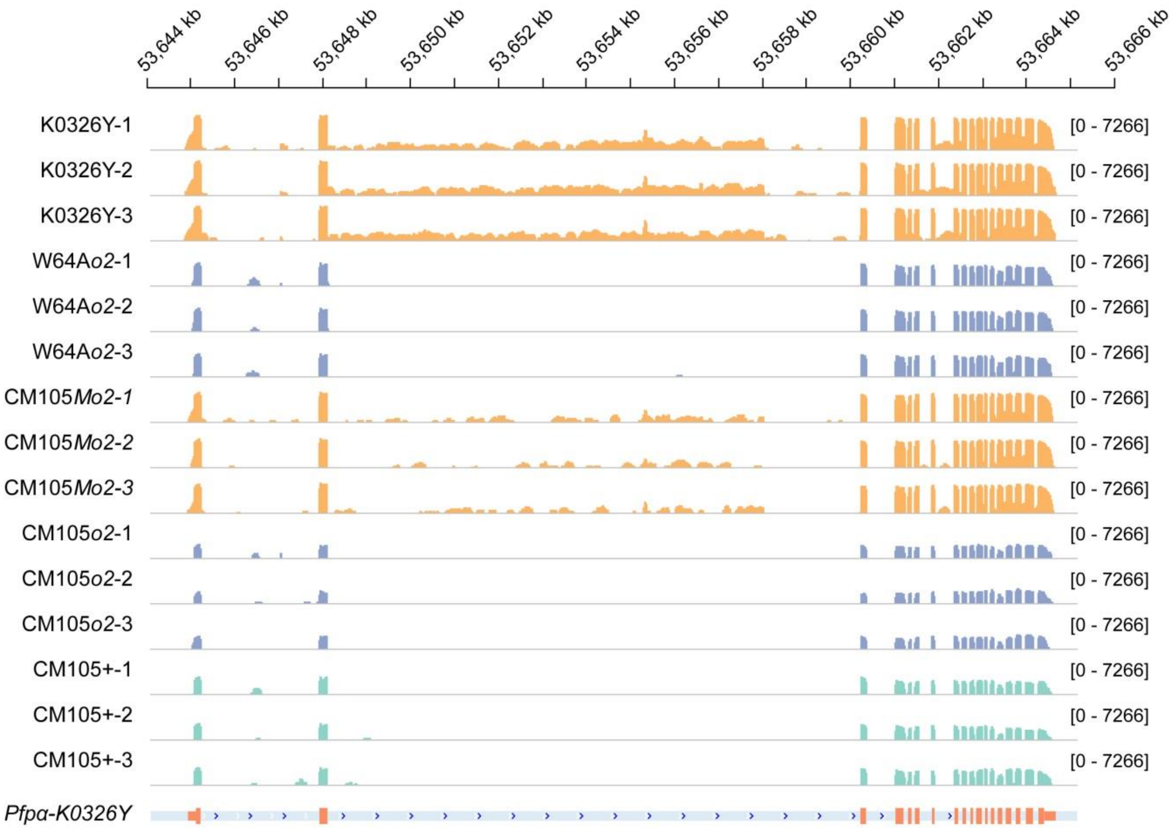

Supplementary Fig. 17. Expression level of *Pfpα* exons. QPMs: K0326Y, CM105M*o2*. Non-QPMs*:* W64A*o2*, CM105*o2* and CM015+. Each inbred line has three biological replicates. The last track is the annotation of *Pfpα* gene with exons in orange and introns in light blue.

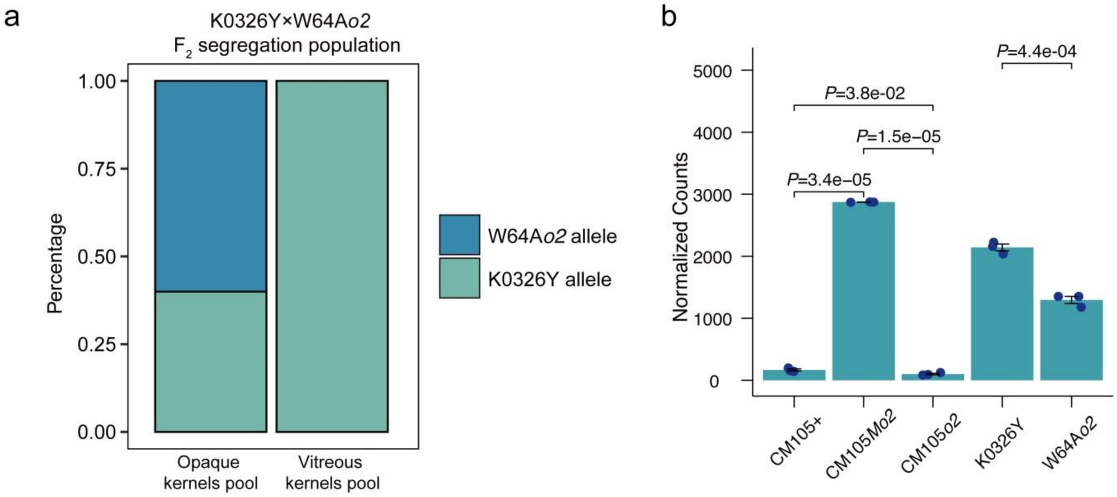

Supplementary Fig. 18. Assocaiation and gene expression of candidate gene of SR45a. (a) Allele frequency of the 399-bp DNA transposon of *hAT* element in the 5th intron of *SR45a* in F_2 segragation population. (b) The gene expression of *SR45a*. The transcript level is measured by using normalized RNA-seq data based on the negative binomial distribution. *P*-values were determined by two-tailed Student's t-test. Error bars were defined as sample standard deviation from the sample size of 3. Source data underlying Supplementary Figure 18b are provided as a Source Data file.

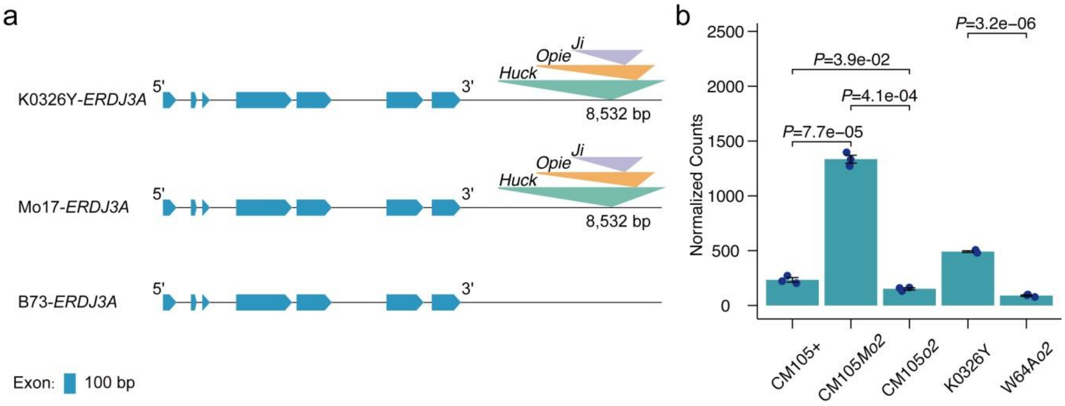

Supplementary Fig. 19. The gene structure and gene expression of *ERDJ3A*. (a) The gene structure of *ERDJ3A* in K0326Y, Mo17 and B73. Exons are colored in blue pentagons and introns are labeled as black lines. The nested retrotransposon with the total size of 26,022 bp is inserted at 8,532 bp. (b) The gene expression of *ERDJ3A*. The transcript level is measured by using normalized RNA-seq data based on the negative binomial distribution. *P*-values were determined by two-tailed Student's t-test. Error bar, SD (n = 3). Source data underlying Supplementary Figure 19b are provided as a Source Data file.

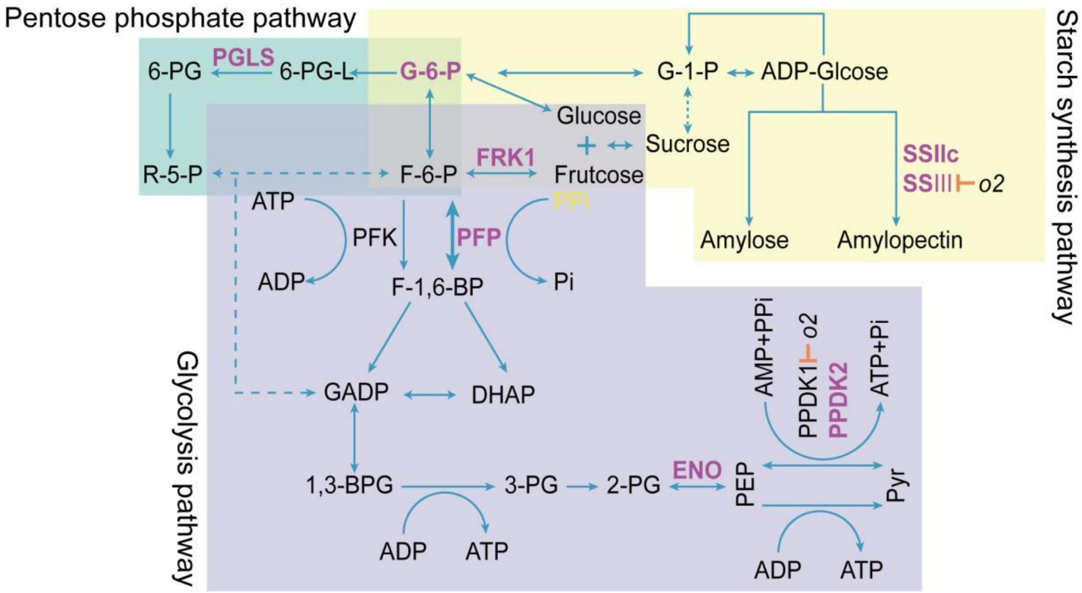

Supplementary Fig. 20. The pathway of starch synthesis, pentose phosphate and glycolysis in QPM. The gene expression was significantly increased labeled in magenta. The mutaion of *O2* can inhibit the expression of PPDK1.

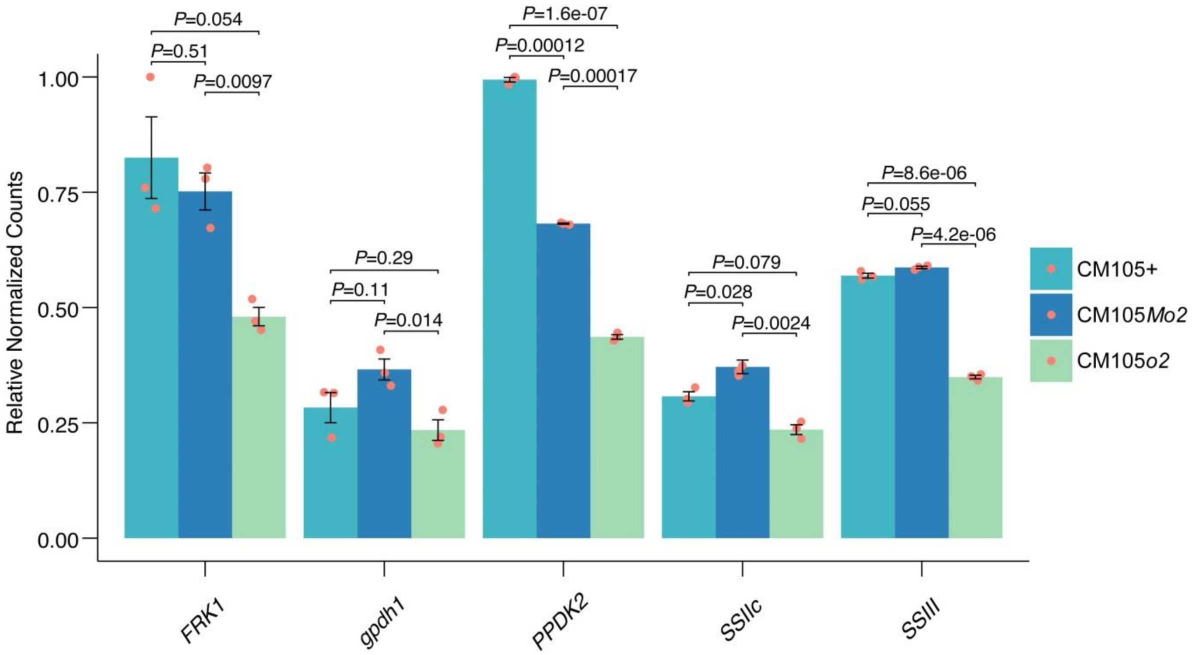

Supplementary Fig. 21. The transcript levels for genes in the pathway of starch synthesis, pentose phosphate and glycolysis in QPM and non-QPM. Abbreviations: *FRK* (*fructokinase*), *gpdh* (*Glucose-6-phosphate*), *PPDK* (*pyruvate Pi dikinase*), *SS (starch synthesis*). *P*-values were determined by two-tailed Student's t-test. Error bars were defined as sample standard deviation from the sample size of 3. Source data are provided as a Source Data file.

Supplementary Table 1. Summary of PacBio read length and genome assembly contig N50.

	B73	Mo17	SK	K0326Y
PacBio Read N50/Mean (kb)	11.7	13.3	12	16.6
Contig N50 (Mb)	1.04	1.48	5.93	6.99

Supplementary Table 2. Summary of sequencing data.

Subreads	Pacbio	Bionano	Illumina
Library	20 kb	100 kb	350 bp
Platform	Sequel	Irys	Hiseq X
Total length (Gb)	299.5	389.3	217.5
**Sequencing depth***	139	180	100
N50 of read length (kb)	16.6	144	-
Longest read (Mb)	0.213	2.68	-
Read number	28,351,764	4,100,737	1,449,884,260

*Estimated with the genome size of 2.16 Gb.

Supplementary Table 3. Statistics for PacBio Sequel sequencing data.

Subreads	Total bases (bp)	Count	**Depth***
>=5 kb	279,010,747,687	18,881,638	129
>=8 kb	254,717,538,166	15,118,218	118
>=10 kb	235,208,471,469	12,946,086	109
>=12 kb	212,921,394,580	10,919,165	99
>=15 kb	173,274,985,926	7,978,586	80
>=16 kb	159,103,111,930	7,064,005	74
>=20 kb	105,035,976,796	4,038,463	49
>=25 kb	56,128,523,776	1,836,802	26
>=30 kb	27,531,123,853	784,509	13
>=35 kb	12,252,033,783	309,431	6
>=40 kb	4,788,956,756	108,403	2

*Estimated with the genome size of 2.16 Gb.

Supplementary Table 4. Read input, preassembly and assembly output.

Read Input:	
Mean Subread Length	16,848 bp
N50 (Subread Length)	18,261 bp
Total Number of sequenced Bases	254,717,538,166 bp
Number of Reads	15,118,218
FALCON Preassembly (daligner):	
Seed length cutoff	15,000 bp
Pre-Assembled Bases	131,417,852,355 bp
Pre-Assembled Reads	9,662,524
Pre-Assembled N50	16,903 bp
Output (FALCON- Arrow-Pilon):	
Number of Polished Contigs	1,221
Max Contig Length	35,542,197 bp
N50 Contig Length	6,998,658 bp
Sum of Congtig Lengths	2148,380,346 bp

Supplementary Table 5. Genome assembly by using long reads and optical map.

	PacBio assembled Contigs		PacBio+BioNano hybrid Scaffolds	
	Length(bp)	Number	Length(bp)	Number
Max length	35,542,197	-	69,245,458	-
N10	18,441,234	10	55,871,734	4
N20	13,771,207	23	41,492,392	9
N30	10,868,101	41	36,142,346	14
N40	8,770,650	63	32,154,888	21
N50	6,998,658	91	27,981,020	28
N60	5,841,938	124	22,620,619	37
N70	4,623,047	165	18,708,447	47
N80	3,454,484	219	14,084,711	60
N90	2,162,836	298	7,996,179	82
Total	2,148,380,346	1,221	2,161,630,014	870

Supplementary Table 6. Statistics of chromosome-level genome assembly.

Chromosome	Size (bp)	Scaffold	Gene
1	309,042,250	28	5,657
2	246,515,396	19	4,493
3	231,712,363	11	3,705
4	248,141,217	18	4,344
5	227,804,939	21	4,408
6	176,415,776	20	3,103
7	182,138,145	13	3,083
8	181,954,665	13	3,503
9	158,024,157	15	2,894
10	150,197,436	13	2,671
Anchored bases	2,111,946,344(97.74%)	171	37,861
Non-anchored bases	48, 746,908(2.26%)	663	377
Total	2,160,693,252	843	38,238

Supplementary Table 7. BUSCO analysis.

	K0326Y		B73		Mo17	
Description	Number	Percent (%)	Number	Percent (%)	Number	Percent (%)
Complete (C)	1,380	95.8	1,384	96.1	1,374	95.4
Complete and single-copy (S)	1,295	89.9	1,303	90.5	1,285	89.2
Complete and duplicated (D)	85	5.9	81	5.6	89	6.2
Fragmented (F)	17	1.2	18	1.3	21	1.5
Missing (M)	43	3	38	2.6	45	3.1
Total	1,440	100	1,440	100	1,440	100

Supplementary Table 8. Comparison of repetitive elements between K0326Y, B73 and Mo17.

Classification		K0326Y		B73		Mo17	
		Length (bp)	Percent (%)	Length (bp)	Percent (%)	Length (bp)	Percent (%)
Class I: Retrotransposon	All	1,634,145,614	77.38	1,645,098,308	77.89	1,643,107,757	77.80
	SINE	1,157,265	0.05	1,070,884	0.05	1,091,730	0.05
	LINE	13,195,636	0.62	12,522,984	0.57	13,730,143	0.65
	LTR-Retrotransposon	1,619,792,713	76.7	1,631,504,440	77.25	1,628,285,884	77.1
	Copia	501,381,213	23.74	531,391,413	25.16	547,614,328	25.93
	Gypsy	917,426,754	43.44	942,641,263	44.63	900,667,175	42.65
	Others	200,984,746	9.52	157,471,764	7.46	180,004,381	8.52
Class II: DNA Transposon	All	99,663,483	4.72	82,796,224	3.87	95,799,909	4.33
	hAT	16,047,246	0.76	13,749,151	0.64	16,417,870	0.74
	MULE	7,172,633	0.34	6,240,091	0.29	7,474,317	0.34
	CMC-EnSpm	51,779,625	2.45	40,710,603	1.9	46,972,979	2.12
	Harbinger	11,562,605	0.55	11,577,033	0.54	13,016,406	0.59
	TcMar-Stowaway	1,503,722	0.07	1,406,999	0.07	1,414,760	0.06
	Helitron	11,597,652	0.55	9,112,347	0.43	10,503,577	0.47
Unclassified	All	10,376,652	0.49	10,738,340	0.51	10,184,062	0.48
	Small RNA	892,552	0.04	667,854	0.03	925,954	0.04
	Satellites	7,586,689	0.36	288,595	0.01	15,601,478	0.74
	Simple repeats	6,954,284	0.33	2,305,987	0.11	7,506,002	0.36
Total Content		1,759,619,274	83.32	1,741,895,308	82.48	1,773,125,162	83.96

Supplementary Table 9. Identification of centromere-related long terminal repeat.

Chromosome	Median (Mb)	Aligned number	95% CIM (Mb)	Size of 95% CIM (Mb)
1	144.06	104	144.01-146.14	2.14
2	103.07	175	102.95-103.19	0.24
3	91.38	161	91.13-91.50	0.38
4	103.98	130	103.66-104.15	0.49
5	107.89	255	107.77-108.00	0.23
6	53.61	94	53.48-53.68	0.2
7	54.93	46	54.06-55.84	1.76
8	57.7	188	57.58-57.89	0.31
9	64.99	103	62.80-65.18	2.38
10	47.51	110	47.43-47.90	0.47

Supplementary Table 10. Identification of CentC.

Chromosome	Location	Interval size (kb)	Copy number
1	143,513,925	420	873
2	104,133,765	110	203
3	88,130,015	2,850	1,275
4	104,418,276	270	289
5	110,196,174	200	351
6	53,306,936	350	413
7	54,073,770	1,890	255
8	58,856,568	330	164
9	65,187,795	250	695
10	47,451,519	580	684

Supplementary Table 11. Summary of PacBio Isoform sequencing in K0326Y, B73 and W64A.

Inbred	Sample source	Platform	Cells	Reads of Insert	Total FL Reads	Non-Redundant FLNC	High quality FLNC	Mapped FLNC	Non-redundant Isoforms
K0326Y	root, stem, seedling, silk, tasssle, ear, seed, endosperm, callus	RS_Sequel & RS_II	52	8,687,787	3,456,976	1,674,620	1,618,691	1,590,828	247,616
W64A	endosperm	RS_II	16	1,455,008	495,619	166,693	-	166,103	135,629
B73	seedling, ear, tassel, pollen, embryo and endosperm	RS_II	47	3,716,604	1,553,692	-	643,330	606,145	111,151

Supplementary Table 12. Statistics of gene model in K0326Y, B73, and Mo17.

	K0326Y	B73	Mo17
Number of gene models	38,238	39,200	38,620
Number of transcripts	60,475	131,318	46,530
Mean exons per gene	5.54	5.23	5.09
Mean CDS length (bp)	1,126	1,177	1,140
Genes supported by ISO-seq with CDS coverage > 90%#	23,783	20,889	-
Genes supported by ISO-seq with CDS coverage > 50%#	26,228	23,101	-
Genes supported by RNA-seq with CDS coverage > 90%#	25,032	21,625	21,136
Genes supported by RNA-seq with CDS coverage > 50%#	29,768	29,759	28,228

Analyzed based on the longest transcript.

Supplementary Table 13. Genomic polymorphism.

	K0326Y against B73	K0326Y against Mo17
SNP	10,205,511	9,655,364
Small InDel	1,397,901	1,458,329

Supplementary Table 14. Summary of large insertions and deletions (>100bp) in K0326Y.

	K0326Y compared with B73				K0326Y compared with Mo17			
	Insertions		Deletions		Insertions		Deletions	
	Number	Percent (%)	Number	Percent (%)	Number	Percent (%)	Number	Percent (%)
3’ UTR	541	2.74	719	1.8	781	2.44	781	1.68
5’ UTR	393	1.99	429	1.07	509	1.59	507	1.09
5’UTR & 3’UTR	66	0.33	70	0.18	11	0.03	7	0.02
Downstream 1Kb	1,248	6.31	2,698	6.76	2,168	6.76	3,085	6.65
Exonic	3,093	15.64	2,919	7.31	3,125	9.74	3,256	7.02
Intergenic	11,417	57.73	26,544	66.47	19,870	61.96	29,873	64.41
Intronic	1,489	7.53	2,956	7.4	2,849	8.88	4,523	9.75
Splicing	85	0.43	199	0.5	124	0.39	355	0.77
Upstream 1Kb	1,333	6.74	3,106	7.78	2,442	7.61	3,718	8.02
Upstream & downstream	113	0.57	291	0.73	192	0.6	276	0.6
Total	19,778	100	39,931	100	32,071	100	46,381	100

The statistics was based on the annotation of B73 and Mo17genome.

Supplementary Table 15. Summary of large insertions and deletions (>100bp) within genes.

	K0326Y compared with B73				K0326Y compared with Mo17			
	Genes with Insertions		Genes with Deletions		Genes with Insertions		Genes with Deletions	
	Number	Percent (%)	Number	Percent (%)	Number	Percent (%)	Number	Percent (%)
3’ UTR	535	8.41	707	6.76	787	8.44	776	6.23
5’ UTR	6	0.09	414	3.96	507	5.44	502	4.03
Exonic	3,116	49.01	3,648	34.87	3,058	32.8	3,728	29.93
Intronic	1,271	19.99	2,346	22.42	2,402	25.76	3,436	27.59
Splicing	83	1.31	195	1.86	123	1.32	347	2.79
Upstream 1Kb	1,347	21.19	3,153	30.13	2,446	26.24	3,667	29.44
Total	6,538	100	10,463	100	9,323	100	12,456	100

The statistics was based on the annotation of B73 and Mo17 genome.

Supplementary Table 16. Summary of duplicated genes in K0326Y genomes, B73 and Mo17.

	K0326Y	B73	Mo17
Singleton	5,360	5,245	6,453
Dispersed	16,777	13,511	8,645
Tandem	3,842	3,406	2,982
WGD/segmental	12,259	17,038	20,540
Total	38,238	39,200	38,620
