# Supplementary material for: Long-read sequencing reveals genomic structural variations that underlie creation of quality protein maize
Source: Nat Commun. 2020 Jan 7;11:17. doi: 10.1038/s41467-019-14023-2 (PMC6946643; doi:10.1038/s41467-019-14023-2)
Supplement: Supplementary file 3 — Reporting Summary [file 41467_2019_14023_MOESM3_ESM.pdf]

nature research

Corresponding author(s): DBPR,NCOMMS-19-24080B

Last updated by author(s): Dec 10, 2019

Reporting Summary

Nature Research wishes to improve the reproducibility of the work that we publish. This form provides structure for consistency and transparency in reporting. For further information on Nature Research policies, see Authors & Referees and the Editorial Policy Checklist.

Statistics

For all statistical analyses, confirm that the following items are present in the figure legend, table legend, main text, or Methods section.

n/a	Confirmed	
☐	☒	The exact sample size (*n*) for each experimental group/condition, given as a discrete number and unit of measurement
☐	☒	A statement on whether measurements were taken from distinct samples or whether the same sample was measured repeatedly
☐	☒	The statistical test(s) used AND whether they are one- or two-sided<br>*Only common tests should be described solely by name; describe more complex techniques in the Methods section.*
☐	☒	A description of all covariates tested
☐	☒	A description of any assumptions or corrections, such as tests of normality and adjustment for multiple comparisons
☐	☒	A full description of the statistical parameters including central tendency (e.g. means) or other basic estimates (e.g. regression coefficient) AND variation (e.g. standard deviation) or associated estimates of uncertainty (e.g. confidence intervals)
☐	☒	For null hypothesis testing, the test statistic (e.g. *F*, *t*, *r*) with confidence intervals, effect sizes, degrees of freedom and *P* value noted<br>*Give P values as exact values whenever suitable.*
☒	☐	For Bayesian analysis, information on the choice of priors and Markov chain Monte Carlo settings
☒	☐	For hierarchical and complex designs, identification of the appropriate level for tests and full reporting of outcomes
☐	☒	Estimates of effect sizes (e.g. Cohen's *d*, Pearson's *r*), indicating how they were calculated

Our web collection on statistics for biologists contains articles on many of the points above.

Software and code

Policy information about availability of computer code

Data collection	no software was used
Data analysis	R (v3.6) is used in all statistical analysis; Falcon (falcon_kit-0.7) is used to assemble genome; Arrow (smrtlinkrelease_5.0.0.6792) and Pilon (v1.22) are used to correct genome assembly; PBJelly (PBSuite_15.2.20) is used for gap filling; IrysVeiw (v2.5.1) and IrysSolve (v3.1.0) are used to assemble Bionano data; Blasr is used for long reads mapping; BWA-MEM and SOAP2 are used for Illumina paired-end reads and GBS tags mapping; NUCmer (MUMmer v3.1) is used for pseudomolecules construction, genome alignment and PAV analysis; RepeatModeler (v1.0.10) and RepeatMasker (v4.0.7) are used to Repetitive annotation; LTRharvest (v4.4.7) and LTRdigest (v4.4.7) are used to predict the full long terminal repeats (LTR) retrotransposons; SiLix, Vsearch, MAFFT and RAxML are used for family clustering and evolutionary dynamics of retrotransposition; MAKER-P (v2.31.8) are used for gene annotation; BLASTN and TRF (V 4.07b) are used for Identification of centromeric regions ; SMRTlink (v5.0), GMAP (2016-11-07) and ToFU are used for ISO-Seq analysis; Trinity (v2.0.6), TIGR, hisat2 (v2.0.4), htseq-count and DEseq2 are used for RNA-seq analysis; ANNOVAR program is use for variants annotation; MCScanX package is used for identification of duplicated genes; NGMLR, Sniffles, and BEDTools are used for structural variation analysis.

For manuscripts utilizing custom algorithms or software that are central to the research but not yet described in published literature, software must be made available to editors/reviewers. We strongly encourage code deposition in a community repository (e.g. GitHub). See the Nature Research guidelines for submitting code & software for further information.

Data

Policy information about availability of data

All manuscripts must include a data availability statement. This statement should provide the following information, where applicable:

- Accession codes, unique identifiers, or web links for publicly available datasets
- A list of figures that have associated raw data
- A description of any restrictions on data availability

All datasets reported in this study have been deposited in GenBank (NCBI) with the following accession IDs: Genome assembly, VAUS00000000 [https://

www.ncbi.nlm.nih.gov/nuccore/CM018593.1]; Raw data for genome assembly and annotation, PRJNA539996 [https://www.ncbi.nlm.nih.gov/bioproject/PRJNA539996]; Raw data for Bulked Segregant Analysis, PRJNA578235 [https://www.ncbi.nlm.nih.gov/bioproject/PRJNA578235]; and RNA-Seq of QPM and o2 maize,
PRJNA578012 [https://www.ncbi.nlm.nih.gov/bioproject/PRJNA578012]. Data supporting the findings of this work are available within the paper and its Supplementary Information files. A reporting summary for this Article is available as a Supplementary Information file. The datasets generated and analyzed during the current study are available from the corresponding author upon request. The source data underlying Figures 2 and 4, as well as Supplementary Figures 2, 5, 9, 10, 13, 18, 19, and 21 are provided as a Source Data file.

Field-specific reporting

Please select the one below that is the best fit for your research. If you are not sure, read the appropriate sections before making your selection.

☒ Life sciences ☐ Behavioural & social sciences ☐ Ecological, evolutionary & environmental sciences

For a reference copy of the document with all sections, see nature.com/documents/nr-reporting-summary-flat.pdf

Life sciences study design

All studies must disclose on these points even when the disclosure is negative.

Sample size	For genome sequencing, no sample size calculation was performed, as we choose only one maize inbred lines to sequence and assemble. To quantify gene expression by using RNA-seq, we sequenced three biological replicates for five inbreds (sample size, n=15); To map the potential QTL loci by using QTL-seq, we sampled vitreous (sample size, n=160) and opaque kernels (sample size, n=160).
Data exclusions	n/a
Replication	n/a
Randomization	n/a
Blinding	Blinding was not relevant for this study.

Reporting for specific materials, systems and methods

We require information from authors about some types of materials, experimental systems and methods used in many studies. Here, indicate whether each material, system or method listed is relevant to your study. If you are not sure if a list item applies to your research, read the appropriate section before selecting a response.

Materials & experimental systems

n/a	Involved in the study	
☒	☐	Antibodies
☒	☐	Eukaryotic cell lines
☒	☐	Palaeontology
☒	☐	Animals and other organisms
☒	☐	Human research participants
☒	☐	Clinical data

Methods

n/a	Involved in the study	
☒	☐	ChIP-seq
☒	☐	Flow cytometry
☒	☐	MRI-based neuroimaging
