# Supplementary material for: Long-read sequencing reveals genomic structural variations that underlie creation of quality protein maize
Source: Nat Commun. 2020 Jan 7;11:17. doi: 10.1038/s41467-019-14023-2 (PMC6946643; doi:10.1038/s41467-019-14023-2)
Supplement: Supplementary file 4 — Description of Additional Supplementary Files [file 41467_2019_14023_MOESM4_ESM.docx]

**Description of Additional Supplementary Files**

File name: Supplementary Data 1
Description: Summary of PAV genes in K0326Y

File name: Supplementary Data 2
Description: Differentially expressed genes between vitreous and opaque kernels

File name: Supplementary Data 3
Description: Differentially expressed genes associated with heat shock proteins and related genes

File name: Supplementary Data 4
Description: Identification of candidate genes generated from BSA-seq, RNA-seq and genomic variation
