# Supplementary material for: Long-read sequencing reveals genomic structural variations that underlie creation of quality protein maize
Source: Nat Commun. 2020 Jan 7;11:17. doi: 10.1038/s41467-019-14023-2 (PMC6946643; doi:10.1038/s41467-019-14023-2)
Supplement: Supplementary file 2 — Peer Review [file 41467_2019_14023_MOESM2_ESM.pdf]

Reviewers' comments:

Reviewer #1 (Remarks to the Author):

Comments for the manuscript entitled “Introgressions in contiguous chromosomal sequences linked to maize endosperm traits.”

Identifying o2 modifiers (Mo2s) is important to the nutritional quality of maize. The authors assembled a tropical maize inbred QPM and tried to clone Mo2S, which are probably absent in elite maize inbred lines. The manuscript consists of two major sections, genome assembly of QPM and genetic mapping of Mo2S. My general impression is that the first section provided another high-quality maize genome for the research community, which will be useful. However, despite the importance of cloning Mo2S, it seems that the whole second section is pretty much just guessing Mo2S, where solid pieces of evidence were not provided. My other concerns are as follows.

Major:
1. Line 198. There were 4 QTLs identified, but the shape of the newly identified QTL on chromosome 6 was too sharp, which might be a false positive because crossovers in a F2 populations should be very limited.

2. Line 214. Why did Mo2S have to be elevated expressed? The genes with down-regulated expression levels could also be the candidates of Mo2S.

3. Line 214 to 268, I did not see solid pieces of evidence supporting those candidates of Mo2S.

4. “Introgressions” are in the title of the manuscript, but I did not find introgression analysis in the results.

Minor:
1. Line 43. Sequence differences between two paralogous repeats should not be called polymorphisms. Polymorphisms are orthologous sequence differences within a species.

2. Line 140. Less syntenic regions between K0326Y and B73 or Mo17 than those of between B73 and Mo17 are probably has nothing to do with climatic regions and soil conditions. It is more likely due to the intensified divergent selection between B73 and Mo17 for heterosis between the two heterotic groups.

3. Line 206. I do not think chaperone binding, unfolded protein binding, protein folding and response to heat and temperature stimulus are related to vitreous endosperm. Only 4 QTLs, probably representing 4 genes, were found for the vitreous endosperm. However, there were nearly 2,000 DEGs, which are unlikely to represent gene functions of the 4 candidate genes.

4. The language of the manuscript can be improved further.

Reviewer #2 (Remarks to the Author):

The paper describes the genome sequencing of a Quality Protein Maize (QPM) line and highlights the advances compared to sequencing of previous maize genomes. It also integrates a QTL analysis, to identify regions containing candidate opaque-2 modifier genes and structural analysis and expression analysis of candidate genes within the QTLs.
The following is a list of minor and major points that need to be addressed, in order of occurrence not significance. The most significant issues, about the interpretation of the data in the discussion, are

described at the end.

The title could better reflect the main thrust of the paper which is the new genome sequence. The current title is vague and neither refers to the technical advances or QPM. There is no continuity between the main title and the running title

Line 32. "Maize is an example" is not a sentence

Line 35. "compared to previously sequenced maize genomes" is not valid because no comparison is made.

Line 36. "key to breeding nutritional quality" is not correct. The modifiers are key to breeding endosperm vitreousness whereas the o2 allele alone specifies nutritional quality.

Line 37. "we identified QTLs with structural variations that explain the molecular basis of vitreous endosperm in QPM" The QTLs are suggestive. They don't explain the vitreousness.
Abstract in general should articulate why this genome sequencing is unique and an advance on previously sequenced maize genomes.

Line 44. "Therefore, a new platform with long-reads from single DNA molecules can bring an answer to complex plant genomes". 'Bring an answer' makes this sentence meaningless

Line 48-49. The analysis itself does not affect allelic diversity.

Line 52. "The reason for this is that the most abundant storage proteins in maize endosperm" The reason for what?

Line 58. "transport and storage of large quantities" of what?

Line 63-65. Yes, gamma zein is a major factor but need to mention here and cite that it's the larger number of smaller protein bodies that is key here

Line 68. What is meant by "uncover such introgressions"

Line 174-177 where did the expression comparison between QPM and o2 spring from? This is not part of the abstract and one has to go to the methods to find out that RNA seq was done. The overlaying of QPM vs wt sequence, mapping by BSA, allelic diversity and differential expression of candidate modifiers are the central important principle of the study yet it is not referred to at all in the abstract or introduction. Both the abstract and introduction need reworking to emphasize the thrust of the study.

Line 175. Biosynthesis not biosynthetic

181-187 talks about differences in gene duplications between B73/Mo17 and K0326Y QPM. I looked for and did not find discussion about the significance of this

190-192. An F2 populations was made from the W64Ao2 x K0326Y cross for the purposes of a BSA analysis. It should be mentioned and cited up from that this is a repeat of a previous mapping population in the 2008 and 2011 papers that were cited elsewhere in the paper

199-201 "vitreous and opaque pools" presumably these RNAs were made from developing kernels and some methodological detail is needed here so that the reader doesn't have to go to the methods section to understand the significance of what was done

Line 204-207. “The increased DEGs were significantly enriched in the terms of chaperone binding, unfolded protein binding, protein folding and response to heat and temperature stimulus (Supplementary Fig. 13), indicating their potential impact on the formation of vitreous endosperm.” This speculative statement is out of the blue and is completely without basis.

Line 207-210. This is much more relevant and should be elaborated upon.

Line 213-215. As already noted, this strategy needs to be emphasized much earlier on.

Line 226. Duplication not duplicate

Line 215-225. A deletion in the promoter of the O10 gene in QPM is shown as is an increase in O10 expression in QPM. However in lines 222-225, it is not clear what conclusion is being drawn about this potential candidate gene.

Line 226-237 talks about the previously described duplication in the 27kD gamma zein gene. But emphasis needs to be made about what new is learned about this proven o2 modifier.

Lines 238-239. This analysis did not “surface” another candidate gene (implying discover). PFPalpha was discovered and extensively described as a candidate gene in three other papers cited elsewhere in this paper. The increased expression and protein content was mentioned as agreeing with previous works at the end of this paragraph. As the authors are aware, the 2012 plant physiology paper was exclusively devoted to analysis of this candidate gene including much of the theories about its role in mediating a hypothetical ATP shortage, that are presented as new theories in the discussion of the current manuscript. The three papers need to be cited as a first mention of PFP alpha followed by a summary of what was already found/hypothesized. Any results and discussion presented about PFP need to build off these works.

Line 242. The new data about PFP alpha are mainly the structural differences and this needs better emphasis.

Line 251-3. As mentioned above, the references should be cited and discussed up front not at end. Also, reference 2 is not correct. It does not mention PFP alpha. The correct refs are 7,8 and 23.

Figure 1. Why is figure 1 showing degrees of modification in pool 42? What is pool 42? Wouldn’t showing kernels of B73, MO17, o2, K0326YQPM and CM105QPM be more useful to the reader…or at least as well as what is currently shown? Also the figure need better annotation.

Discussion.
The discussion needs completely re thinking and re writing.
Line 271-282. As inferred from the running title, the main point of the paper is the sequencing of a QPM genome. There is essentially no discussion of the sequencing innovations apart from a few lines at the beginning of the discussion. This needs to be discussed extensively and become the main focus of the discussion.

Most of the discussion about candidate genes is way over speculative and much of it is based on unacknowledged theories presented by a different group in a 2012 Plant Physiology paper. This discussion needs to carefully interpret any new insights the current data bring and put it in a proper context of past data and theories.

As stated above, the discussion of PFP alpha, PPDK and chaperones should build off and cite results and interpretations previously published, not ignore them.

Line 287-289 “Noticeably, the inner maize endosperm environment is anoxic, and ATPs are generated

mainly from glycolysis rather than the tricarboxylic acid (TCA) cycle (Supplementary Fig. 20)” This statement is based off theories offered in the previous 2012 plant physiology paper which cites the original work by Rolletschek et al that describes increasing hypoxia in the central endosperm. Neither paper has been cited here. It is not “noticeable”. It has not been measured here and it cannot be stated that this leads to ATP coming mainly from glycolysis which is speculation but presented as fact.

Line 289-293. Again this is speculative but presented as fact.

Line 293-294. This is not a novel idea. It was presented in the Plant Physiology paper and it is speculative.

Line 294-296. PPDK is a bi-directional glycolytic enzyme that can also act in the gluconeogenic direction. Therefore it is not possible to speculate on the effect of its reduced gene expression glycolytic flux or on ATP supply.

295-297. This is speculative and without reason about the effects of starch on vitreousness. All we know for sure is the effect of altered zein abundance and protein body number, shape and size on vitreousness.

Line 306. No mention is made that PFPalpha is the regulatory subunit of a heterotetrameric holoenzyme in which PFP beta is the catalytic subunit.

Line 308-309 The idea that QPM uses an altered energy currency as part of the recovery of vitreous endosperm is not new but was extensively described before. Indeed, a curtailed description is presented which does not provide any new evidence for this theory beyond speculation

Line 309-311. “Partially restored ATP levels” This was not measured so this speculation is not valid

The model presented ignores previous published data showing that the expression of many HSPs, that are elevated in o2, are returned to normal wild type expression levels in QPM endosperm and thus, that UPR is likely reduced in QPM endosperm compared with o2. The model presented implies that UPR stress is further increased in QPM endosperm which would be the opposite of what was previously shown.

Dear Editor and Reviewers:

Thank you for your review and comments concerning our manuscript entitled "Long-read sequencing reveals genomic structural variations that underlie creation of Quality Protein Maize". We appreciate your concerns and suggestions. The comments are all valuable and very critical to improve our manuscript. We have revised our manuscript accordingly and have made necessary correction, which we have tracked in the manuscript. We specifically highlighted the prior knowledge about QPM, our sample preparation and the functions of heat shock proteins. We also explain the discrepancy between our analysis and previous study.

Reviewers' comments:

Reviewer #1 (Remarks to the Author):

Comments for the manuscript entitled "Introgressions in contiguous chromosomal sequences linked to maize endosperm traits."

Identifying o2 modifiers (Mo2s) is important to the nutritional quality of maize. The authors assembled a tropical maize inbred QPM and tried to clone Mo2S, which are probably absent in elite maize inbred lines. The manuscript consists of two major sections, genome assembly of QPM and genetic mapping of Mo2S. My general impression is that the first section provided another high-quality maize genome for the research community, which will be useful. However, despite the importance of cloning Mo2S, it seems that the whole second section is pretty much just guessing Mo2S, where solid pieces of evidence were not provided. My other concerns are as follows.

Major:

1. Line 198. There were 4 QTLs identified, but the shape of the newly identified QTL on chromosome 6 was too sharp, which might be a false positive because crossovers in a F2 populations should be very limited.

Response: Thank you for the suggestion. I agree with you due to the lack of further examination. We have rephrased this part: "A novel QTL with a sharp peak was found at a region on chromosome 6, with a large K0326Y-specific fragment insertion (Supplementary Fig. 3). Whether it is a false positive or not needs a further examination, because crossovers in a F2 population are very limited. "

2. Line 214. Why did Mo2S have to be elevated expressed? The genes with down-regulated expression levels could also be the candidates of Mo2S.

Response: Thank you for the comment. Yes, both can be candidate *Mo2*s. We summarized 216 DEGs overlapped with QTL regions, including 117 up- and 99 down-regulated genes. However, the most promising candidates, such as *Pfpα*, *SR45a* and *ERDJ3A*, happened to be the genes with elevated expression. We didn't find down-regulated genes with sequence variations that are linked to QTL loci. We added our explanation into text.

3. Line 214 to 268, I did not see solid pieces of evidence supporting those candidates of Mo2S.

Response: Thanks for the comment. The main aim of this study is to provide a reference genome for QPM breeding. We have achieved a high-quality QPM genome sequence and investigated

different kinds of genomic variations compared to B73 and Mo17, some of which should underlie successful breeding of QPM. We then did the *Mo2* QTL mapping by BSA sequencing and analyzed the candidate genes with structural variations and differential expression. Although we did not do genetic validation and functional analysis of these candidate genes, the information we presented here is useful and valuable for future molecular study. Unveiling the mechanism of each *Mo2* in detail could be an independent story in the following research.

4. "Introgressions" are in the title of the manuscript, but I did not find introgression analysis in the results.
Response: As suggested by the reviewer, the tittle has been changed to a more specific one: Long-read sequencing reveals genomic structural variations that underlie creation of Quality Protein Maize.

Minor:
1. Line 43. Sequence differences between two paralogous repeats should not be called polymorphisms. Polymorphisms are orthologous sequence differences within a species.
Response: Thanks for the comment. We haved changed "polymorphisms" into "sequence variations".

2. Line 140. Less syntenic regions between K0326Y and B73 or Mo17 than those of between B73 and Mo17 are probably has nothing to do with climatic regions and soil conditions. It is more likely due to the intensified divergent selection between B73 and Mo17 for heterosis between the two heterotic groups.
Response: Thanks for the comment. We searched the papers for maize breeding history. It is right that it is more likely due to the intensified divergent selection between B73 and Mo17 for heterosis between the two heterotic groups. In the mid 1960's, scientists selected B73, B37, B14 et. al from BSSS (Iowa Stiff stalk synthetic and their derivate), as female parents that could produce enough seeds, but with short period of pollen dispersion. Other inbreds (C103, C123, MO17), that combined well with BSSS became the male pool (J.C. Reif , A.R. Hailauer , A.E. Melchinger, Heterosis and heterosis and heterotic patterns in maize, Maydica,2005). The parental inbreds have been artificially selected for maximum combining ability derived from genomic variation and gene expression differentiation, allowing for the tendency of a crossbred individual to present the hybrid vigor (Zachary B. Lippman , Dani Zamir, Heterosis: revisiting the magic, Trends in Genetics,2007). The development of B73-Mo17 near-isogenic lines illustrated abundant structural variation in the two inbreds (Eichten, S. R , et.al., B73-Mo17 Near-Isogenic Lines Demonstrate Dispersed Structural Variation in Maize, 2011). We have corrected the sentence in the manuscript.

3. Line 206. I do not think chaperone binding, unfolded protein binding, protein folding and response to heat and temperature stimulus are related to vitreous endosperm. Only 4 QTLs, probably representing 4 genes, were found for the vitreous endosperm. However, there were nearly 2,000 DEGs, which are unlikely to represent gene functions of the 4 candidate genes.
Response: Thank you for the comment. It could be true that 4 QTLs represent 4 causal genes. We also believe that most of the identified 1,791 differentially expressed genes (DEGs) (QPM compared to *o2* mutants) could be co-regulated genes derived from the causal signal of *o2*

modifiers. The gene enrichment can provide clues of signaling pathways and network modules. For example, a causal gene of transcription factor can usually regulate different sets of target genes. To clarify the misunderstanding, we have rephrased the sentences:

"DEGs that do not map to QTLs might not necessarily be identified as causing the QPM phenotype, but they can provide clues as to an effect of *Mo2*s that set off a signaling cascade, leading to formation of vitreous endosperm. They could also be under regulation of *Mo2*s, reflecting downstream effects of changes in kernel composition. There were 216 (117 up-regulated and 99 down-regulated) DEGs that cross-checked with genes from the QTL-seq analysis, narrowing the pool of candidate genes contributing to a vitreous kernel phenotype and environmental adaption (Supplementary Table 20). After sorting the 216 gene list, there were 125 containing SNPs, 44 with insertions and 43 with deletions, compared to B73, as well as 18 exhibiting tandem duplications, and 19 belonging to PAVs (Supplementary Table 20).
"

4. The language of the manuscript can be improved further.
Response: As suggested by the reviewer, the language of this manuscript has been comprehensively edited.

Reviewer #2 (Remarks to the Author):

The paper describes the genome sequencing of a Quality Protein Maize (QPM) line and highlights the advances compared to sequencing of previous maize genomes. It also integrates a QTL analysis, to identify regions containing candidate opaque-2 modifier genes and structural analysis and expression analysis of candidate genes within the QTLs.
The following is a list of minor and major points that need to be addressed, in order of occurrence not significance. The most significant issues, about the interpretation of the data in the discussion, are described at the end.

The title could better reflect the main thrust of the paper which is the new genome sequence. The current title is vague and neither refers to the technical advances or QPM. There is no continuity between the main title and the running title.
Response: As suggested by the reviewer, the tittle has been changed to a more specific one: Long-read sequencing reveals genomic structural variations that underlie creation of Quality Protein Maize.

Line 32. "Maize is an example" is not a sentence
Response: We have deleted this short sentence.

Line 35. "compared to previously sequenced maize genomes" is not valid because no comparison is made.
Response: We have rephrased the sentence: We achieve an N50 contig length of 7.7 million bases (Mb) directly from long-read assembly, compared to those of 1.04 Mb for B73 and 1.48 Mb for Mo17.

Line 36. "key to breeding nutritional quality" is not correct. The modifiers are key to breeding endosperm vitreousness whereas the o2 allele alone specifies nutritional quality.

Response: Thank you for the correction. We have deleted the sentence in this place.

Line 37. “we identified QTLs with structural variations that explain the molecular basis of vitreous endosperm in QPM” The QTLs are suggestive. They don’t explain the vitreousness. Abstract in general should articulate why this genome sequencing is unique and an advance on previously sequenced maize genomes.
Response: Thanks for the suggestions. We have rephrased the sentences and emphasized the uniqueness and significance of sequencing QPM: “The maize *o2* mutation doubles the endosperm lysine content, but it causes an inferior kernelp henotype. Creation of Quality Protein Maize (QPM), by introgressing *o2 modifiers* (*Mo2s*), has benefit millions of people in developing countries where maize is a primary protein source.”

Line 44. “Therefore, a new platform with long-reads from single DNA molecules can bring an answer to complex plant genomes”. ‘Bring an answer’ makes this sentence meaningless
Response: Thanks for the correction. We have rephrased the sentence into a more specific way: “A new platform with long reads from single DNA molecules can overcome the low resolution of reconstructed repetitive regions in complex plant genomes.”

Line 48-49. The analysis itself does not affect allelic diversity.
Response: Thanks for the suggestion. We have corrected it into “demonstrating”.

Line 52. “The reason for this is that the most abundant storage proteins in maize endosperm” The reason for what?
Response: We have rephrased the sentence as follows: “It is well known that the most abundant storage proteins in maize endosperm, prolamins called zeins, are deficient in the essential amino acids, lysine and tryptophan, requiring costly dietary supplementation.”

Line 58. “transport and storage of large quantities” of what?
Response: We have rephrased the sentence as follows: “However, the chalky and soft endosperm texture of *o2* impairs the transport and storage of the grain, and facilitates fungal infection and insect susceptibility.”

Line 63-65. Yes, gamma zein is a major factor but need to mention here and cite that it’s the larger number of smaller protein bodies that is key here
Response: We have mentioned gamma zein as a major *Mo2* and cited the papers as follows: “It was shown that the 27-kD γ-zein (γ27), a cysteine-rich protein, is an important factor for endosperm modification in QPM[7]. This protein plays a role in the initiation and stabilization of zein protein bodies (PBs), allowing them to accumulate in the rough endoplasmic reticulum[8,9]. *qγ27*, a major *Mo2*, resulted from a gene duplication at the *γ27* locus. The increased *γ27* gene copy number elevates both transcript and protein levels, leading to more PBs that form around starch granules. These PBs are smaller than wild type, due to the *o2* mutation causing a reduction of α-zeins[7,10-15].”

Line 68. What is meant by “uncover such introgressions”
Response: We have rephrased it as follows: “To characterize these loci, we sequenced a QPM genome (K0326Y) and compared it with B73 and Mo17 genomic variations, including single

nucleotide polymorphisms (SNPs), insertions and deletions (InDels), presence and absence variations (PAVs), and other structural variations."

Line 174-177 where did the expression comparison between QPM and o2 spring from? This is not part of the abstract and one has to go to the methods to find out that RNA seq was done. The overlaying of QPM vs wt sequence, mapping by BSA, allelic diversity and differential expression of candidate modifiers are the central important principle of the study yet it is not referred to at all in the abstract or introduction. Both the abstract and introduction need reworking to emphasize the thrust of the study.
Response: Thanks for the comment. We have emphasized this in end of the introduction:
"To characterize these loci, we sequenced a QPM genome (K0326Y) and compared it with B73 and Mo17 genomic variations, including single nucleotide polymorphisms (SNPs), insertions and deletions (InDels), presence and absence variations (PAVs), and other structural variations. We also conducted a bulked segregation analysis of the F_2 population of K0326Y and W64A*o2* to identify *Mo2* QTLs. We identified 1,791 differentially expressed genes (DEGs) from RNA-seq analysis of two sets of QPMs and *o2* mutants (K0326Y versus W64A*o2* and CM105*Mo2* versus CM105*o2*). Among these DEGs we found a group of candidate genes tightly linked to *Mo2* loci, with genomic structural variations and altered expression in QPMs. Based on these and previous studies, we propose a model explaining the formation of vitreous endosperm in QPM. We hypothesize that an increased generation of ATP through glycolysis and an enhanced unfolded protein response (UPR) contribute to alleviating an energy deficiency in *o2*, and directly or indirectly facilitate the formation of vitreous endosperm in QPM endosperm. The high-quality QPM genome sequence we created and the candidate genes with structural variations and differential expression we identified will be useful for future molecular characterization and creation of QPMs."

Line 175. Biosynthesis not biosynthetic
Response: We have corrected the word.

181-187 talks about differences in gene duplications between B73/Mo17 and K0326Y QPM. I looked for and did not find discussion about the significance of this
Response: We have discussed this in detail.
"Analysis of maize genome intraspecific variation has important implications for crop improvement and plant breeding. Sequencing an entire genome allowed us to reveal the degree of copy number variation and presence/absence variation in maize, which is frequently associated with a change in gene expression and contributes to extraordinary phenotypic diversity[28]. In this study, we identified a high level of gene duplications in the K0326Y genome, including dispersed (16,777) and tandem (3,824) duplicated genes. We also uncovered 39,479 segments present and 631 PAV genes with differential expression in K0326Y, but absent in B73, indicating potential impact on phenotypic diversity and underlying mechanism of heterosis. There are more than 40 gene copies accounting for the abundant α-zeins that comprise the majority of seed storage proteins in maize[38,39]. The tandem duplications of these genes may be important for breeding, as the expansion and contraction of gene copies could be tied to important quantitative traits. It was shown that a gene duplication conferred enhanced transcript and protein levels of the *γ27* gene, which was proved important for endosperm modification in QPM[10]. The availability of the whole genome allowed us to investigate this and other loci and to

make comparative analyses between QPM and non-QPM genotypes. It is possible that knowledge of genomic variation in maize will provide source material to generate novel alleles. There is evidence that strong artificial selection on specific anthocyanin coloration patterns has often led to the formation of complex alleles with tandem duplications[40]."

190-192. An F2 populations was made from the W64Ao2 x K0326Y cross for the purposes of a BSA analysis. It should be mentioned and cited up from that this is a repeat of a previous mapping population in the 2008 and 2011 papers that were cited elsewhere in the paper.
Response: We have emphasized this and cited the papers in right places.

199-201 "vitreous and opaque pools" presumably these RNAs were made from developing kernels and some methodological detail is needed here so that the reader doesn't have to go to the methods section to understand the significance of what was done
Response: We have added the methods into the text: "To identify candidate genes corresponding to *Mo2* QTLs, total RNA from developing maize endosperm was collected at 16 DAP."

Line 204-207. "The increased DEGs were significantly enriched in the terms of chaperone binding, unfolded protein binding, protein folding and response to heat and temperature stimulus (Supplementary Fig. 13), indicating their potential impact on the formation of vitreous endosperm." This speculative statement is out of the blue and is completely without basis.
Response: Thanks for the comment. We have rephrased this part: "DEGs that do not map to QTLs might not necessarily be identified as causing the QPM phenotype, but they can provide clues as to an effect of *Mo2*s that set off a signaling cascade, leading to formation of vitreous endosperm. They could also be under regulation of *Mo2*s, reflecting downstream effects of changes in kernel composition. There were 216 (117 up-regulated and 99 down-regulated) DEGs that cross-checked with genes from the QTL-seq analysis, narrowing the pool of candidate genes contributing to a vitreous kernel phenotype and environmental adaption (Supplementary Table 20). After sorting the 216 gene list, there were 125 containing SNPs, 44 with insertions and 43 with deletions, compared to B73, as well as 18 exhibiting tandem duplications, and 19 belonging to PAVs (Supplementary Table 20). "

Line 207-210. This is much more relevant and should be elaborated upon.
Response: Thanks for the comment. We have rephrased this part: "To identify candidate genes corresponding to *Mo2* QTLs, total RNA from developing maize endosperm was collected at 16 DAP. The transcriptomic profiles of vitreous and opaque kernels were built to compare gene expression levels between K0326Y and W64A*o2* and between CM105*Mo2* (another QPM line in the CM105 background) and CM105*o2*. There were 1,791 DEGs that overlapped in the two comparisons, of which 926 were up-regulated and 865 down-regulated (Supplementary Table 18). The increased DEGs were significantly enriched in the terms of chaperone binding, unfolded protein binding, protein folding and response to heat and temperature stimulus (Supplementary Fig. 13). Notably, 43 genes encoding heat shock protein (HSPs) and HSP transcription factors were significantly up-regulated in the QPM (Supplementary Table 19). As previously suggested[30], they might be activated and reduce stress effects by reassembling unfolded or aggregated proteins created by the *o2* mutation." We re-drew the supplementary Fig. 11 to make our analysis pipeline clearer (Please see next answer).

Line 213-215. As already noted, this strategy needs to be emphasized much earlier on.

Response: Thanks for the comment. We have added such information in the end of discussion: "To characterize these loci, we sequenced a QPM genome (K0326Y) and compared it with B73 and Mo17 genomic variations, including single nucleotide polymorphisms (SNPs), insertions and deletions (InDels), presence and absence variations (PAVs), and other structural variations. We also conducted a bulked segregation analysis of the F_2 population of K0326Y and W64A*o2* to identify *Mo2* QTLs. We identified 1,791 differentially expressed genes (DEGs) from RNA-seq analysis of two sets of QPMs and *o2* mutants (K0326Y versus W64A*o2* and CM105*Mo2* versus CM105*o2*). Among these DEGs we found a group of candidate genes tightly linked to *Mo2* loci, with genomic structural variations and altered expression in QPMs."

We have also made the pipeline clearer to readers by presenting a revised Supplementary Fig. 11.

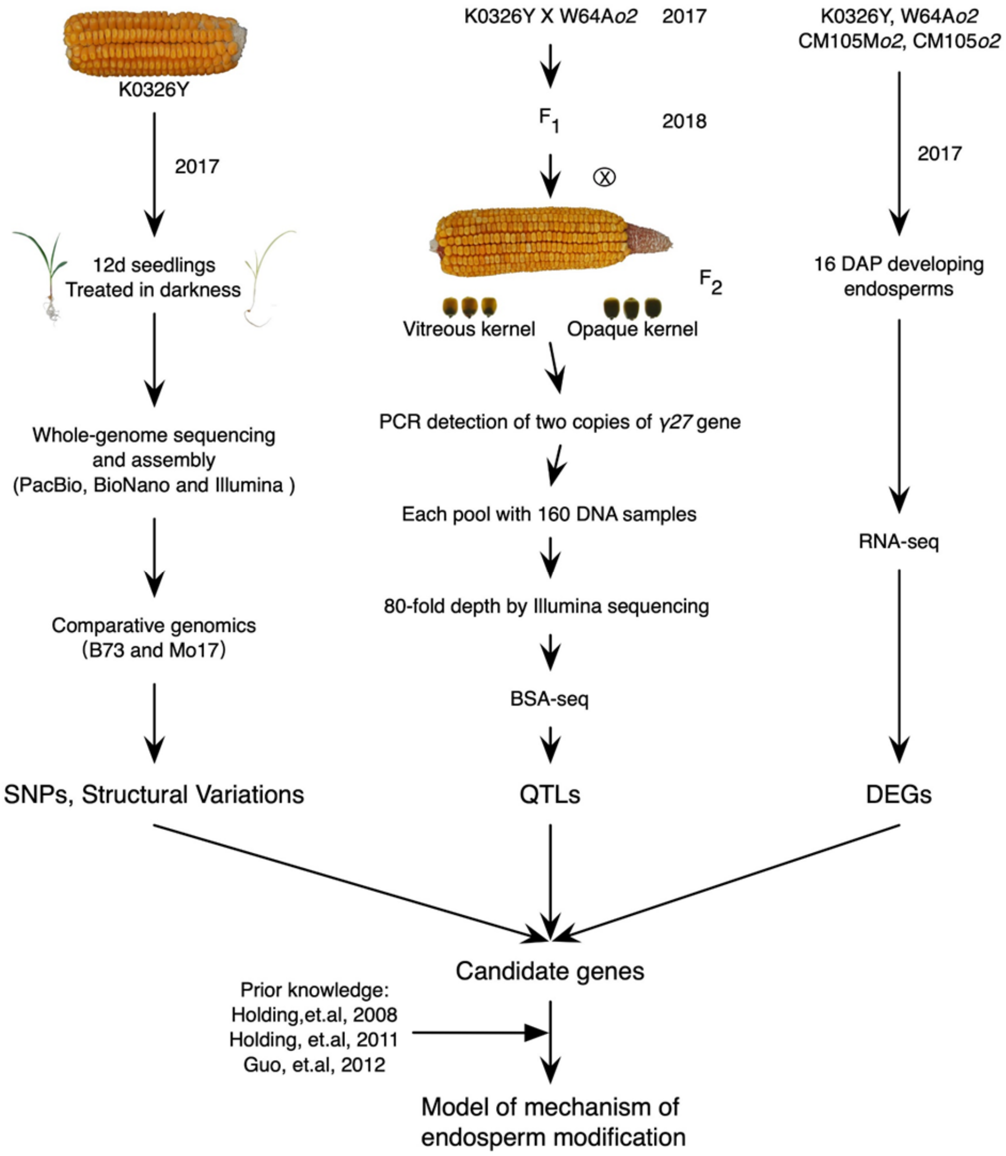

Line 226. Duplication not duplicate
Response: We have corrected the word.

Line 215-225. A deletion in the promoter of the O10 gene in QPM is shown as is an increase in O10 expression in QPM. However in lines 222-225, it is not clear what conclusion is being drawn about this potential candidate gene.
Response: Thanks for the comment. We have rephrased this as follows:
"On chromosome 1, there was 85-bp deleted from the *O10* promoter in K0326Y, which was highly expressed in K0326Y and CM105*Mo2* QPM lines. *O10* encodes a novel cereal-specific protein that regulates zein deposition and organization in PBs[31]. Mutations in *O10* create abnormal distribution of zeins in PBs. Whether up-regulation of *O10* functions as a *Mo2* or is required for *Mo2*-mediated endosperm modification remains to be investigated."

Line 226-237 talks about the previously described duplication in the 27kD gamma zein gene. But emphasis needs to be made about what new is learned about this proven o2 modifier.
Response: Thanks for the suggestion. We have mentioned this in detail:
"Tandem duplication of the *γ27* locus (*qγ27*), which was previously designated the *Standard* allele (*S*) for bearing two copies of *γ27* gene, is a major *Mo2* for endosperm modification in QPM[10]. Besides *γ27*, this duplication involves three other genes (GRMZM2G565441, GRMZM2G138976 and GRMZM5G873335) based on B73_vs3, which were merged into one gene with fifteen exons encoding ARID-transcription factor 4 (Zm00001d020593)[18]. Although all QPM lines have this duplication, a contiguous genomic sequence was not previously available and its variation was unknown[10]. Here, we were able to expand this sequence to 28 kb, totally covering the duplication (~15 kb). The duplicated fragments have many SNPs that could distinguish them. The first copy of *ARID4* has a 1,923-bp deletion in its 3' end, resulting in four missing exons, compared to the second gene copy (Supplementary Fig. 14). A polymorphic PCR primer pair flanking this deletion could be designed to select for the duplication during QPM breeding, depending on the presence of two PCR bands. A global alignment showed that the *qγ27* locus between Mo17 and K0326Y were nearly identical. The B73-*γ27* allele was closer to the first fragment copy in K0326Y, and B73-*ARID4* was closer to the second one in K0326Y (Supplementary Fig. 15). This indicates that the single copy *γ27* allele in B73 could have been produced from DNA rearrangement of the duplicated allele. Therefore, modern maize lines might have had at least three types of single copy *γ27* allele, whereas the tandem duplication might have occurred early before maize domestication (Supplementary Fig. 16). "

Lines 238-239. This analysis did not "surface" another candidate gene (implying discover). PFPalpha was discovered and extensively described as a candidate gene in three other papers cited elsewhere in this paper. The increased expression and protein content was mentioned as agreeing with previous works at the end of this paragraph. As the authors are aware, the 2012 plant physiology paper was exclusively devoted to analysis of this candidate gene including much of the theories about its role in mediating a hypothetical ATP shortage, that are presented as new theories in the discussion of the current manuscript. The three papers need to be cited as a first mention of PFP alpha followed by a summary of what was already found/hypothesized. Any results and discussion presented about PFP need to build off these works.
Response: Thanks for the comment. We have cited these papers and discriminated the previous and the current work. In this study, the advance is that we have achieved high-quality sequence

and found large genomic variations in this gene between QPM and B73 and Mo17. This new finding explained the differential expression of *Pfpα* gene between QPM and non-QPM lines.

Line 242. The new data about PFP alpha are mainly the structural differences and this needs better emphasis.
Response: Thanks for the comment. We have emphasized this in the revised manuscript.
"The identity of the causative polymorphisms and underlying expression mechanisms influencing *Pfpα* had not been elucidated due to the lack of a QPM genome sequence. We found that the *Pfpα* locus in K0326Y showed dramatic structural variations compared to Mo17 and B73. In K0326Y-*Pfpα*, there was a 983-bp *Helitron* in the promotor and a 2,485-bp insertion in the second intron, compared to Mo17-*Pfpα* and B73-*Pfpα*. However, the Mo17-*Pfpα* and B73-*Pfpα* both contained a 6,181-bp CACTA, a 10,685-bp retrotransposon in the second intron, and a 6,037-bp retrotransposon in the 13th intron, compared to the K0326Y-*Pfpα* (Fig. 4a). To investigate whether these insertions and deletions affect *Pfpα* expression, the transcripts of each exon were normalized to the exon size and sequencing depth. We found that the transcript abundance of the whole gene and each exon of *Pfpα* in QPMs (K0326Y and CM105*Mo2*) were higher than non-QPM lines (W64A*o2*, CM105*o2*, CM105+) (Fig. 4b and Supplementary Fig. 17). In addition, we found that 76% of QPM lines have the *Helitron* insertion. The *Helitron* insertion was retained in 95% of the vitreous endosperm kernels of the segregating F_2 population (Fig. 4c), and is therefore clearly associated with the vitreous kernel phenotype. Mobile genetic elements can drive genome evolution, and also alter gene expression by insertion within introns, exons or regulatory region. Transposon-mediated mutations in the promoter of *Adh1* resulted in a significant increase of gene expression in pollen[32]. A transposable element inserted into the regulatory region of a maize domestication gene (*teosinte branched1*, *tb1*) acted as an enhancer of its expression, which partially explained increased apical dominance in maize[33]. The *Helitron* insertion into the promoter of *Pfpα* might be partially causative of endosperm modification in QPM. Whether other transposable elements influence functional variation requires further research."

Line 251-3. As mentioned above, the references should be cited and discussed up front not at end. Also, reference 2 is not correct. It does not mention PFP alpha. The correct refs are 7,8 and 23.
Response: We have corrected this.

Figure 1. Why is figure 1 showing degrees of modification in pool 42? What is pool 42? Wouldn't showing kernels of B73, MO17, o2, K0326YQPM and CM105QPM be more useful to the reader…or at least as well as what is currently shown? Also the figure need better annotation.
Response: We have added the description of Pool42 and the link to the request for this material in the Methods and Materials: "To compare varying degrees of endosperm modification for *o2* mutants, 100-kernels of a record QPM population, O2POOL 42, segregating *Mo2*s were obtained from the International Maize and Wheat Improvement Center, CIMMYT (http://mgb.cimmyt.org/gringlobal/accessiondetail.aspx?id=14778, plant ID 014785). They were grown in Sanya, Hainan."

Figure 1 shows a good comparison of ears from the QPM breeding population with different extents of endosperm modification. For K0326Y, we don't have the WT and non-modified *o2*

control in K0326Y background. For CM105*o2* and CM105*Mo2*, they are totally unmodified or modified. Figure 1 can convey the information how QPM is selected from a population.

Discussion.
The discussion needs completely re thinking and re writing.

Line 271-282. As inferred from the running title, the main point of the paper is the sequencing of a QPM genome. There is essentially no discussion of the sequencing innovations apart from a few lines at the beginning of the discussion. This needs to be discussed extensively and become the main focus of the discussion.

Response: Thanks for the comment. We have expanded the discussion regarding this as following: "Contiguous sequence information achieved by shotgun DNA sequencing[36] and sequencing technologies advances the molecular genetic analysis of complex genomes in plants and animals. The B73 maize genome was improved with a 52-fold increase in contig length and a notable retrieval of intergenic spaces and centromeres by using single-molecule technologies[18]. Long-read sequences not only uncover extensive SNP and InDel variations, but also identify extensive structural variations between maize inbred lines, which is not apparent from genomes assembled with short reads[37]. The tropical SK maize genome generated from long reads presents 80,614 polymorphic structural variants, across 521 diverse lines and serves as a reference genome to mine novel genetic variation for crop improvement[17]. Extended contiguous chromosomal information also provides a new approach to reconstruct breeding histories. The high-quality Mo17 genome sequence was found to contain extensive intraspecific genome diversity compared to other maize lines, which provided the opportunity to explore the molecular basis of heterosis and genome evolution[19]. In particular, traits composed of multiple QTLs with different weights can be assigned to precise chromosomal regions."

"Previous molecular characterization of K0326Y QPM was limited by the absence of a complete genome sequence. The *qγ27* QTL was associated with a major *Mo2* based on linkage mapping[15]. To investigate the structure of *qγ27*, a Mo17 BAC clone containing the duplicated *γ27* gene was sequenced[10], as it would have been extraordinary labor-intensive and time-consuming to determine the structure and expression of any *Mo2* candidate gene in K0326Y without the genome sequence. In this study, we produced a high-quality QPM genome sequence with a contig N50 of 6.99 Mb and captured many sequence polymorphisms and structural variations. We can now trace different introgressions achieved through intensive breeding and thereby uncover multiple potential *Mo2* molecular mechanisms for the development of hard kernel varieties, which can accelerate future breeding via marker-assisted selection.
"

Most of the discussion about candidate genes is way over speculative and much of it is based on unacknowledged theories presented by a different group in a 2012 Plant Physiology paper. This discussion needs to carefully interpret any new insights the current data bring and put it in a proper context of past data and theories.

Response: Thanks for the comment. We have cited the paper in the right place.

“Holding et.al mapped three major *Mo2* QTLs in K0326Y on chromosomes 1, 7 and 9, using two independently developed QPM lines and recombinant inbred lines (RILs)[15,16]. A microarray hybridization identified 16 differentially up-regulated genes in QPM, including *Pfpα* and *γ27*, some of which showed a consistent expression pattern with an increase in vitreous endosperm compared with opaque RILs[15,16]. The PFP protein complex, consisting of two regulatory *α*-subunits and two catalytic β-subunits, is a non-ATP-dependent enzyme that could catalyze a rate-limiting step in glycolysis. It was proposed that increased PFP*α* activity in QPM could attenuate oxygen stress in the endosperm by increasing diminished ATP reserves; PFPβ expression was totally uniform between genotypes[30]. The O2 transcription factor could positively regulated pyruvate orthophosphate dikinase 1 (PPDK1)[41], as this enzyme is down-regulated in *o2* mutants, making it important during late-stage kernel filling. However, an exclusively cytosolic version of PPDK2 that might enhance non-ATP-dependent glycolytic flux was shown to be up-regulated in QPM endosperm[30]. We identified another ATP-independent enzyme, cytosolic enolase (ENO), with increased gene expression in QPM. ENO is an integral enzyme in glycolysis, catalyzing the interconversion of 2-phosphoglycerate to PEP. It was reported that enolase transcript levels and enzyme activity were induced by anaerobic stress in maize[42]. This is consistent with a hypothesis that the increased synthesis of non-ATP-requiring enzymes for glycolysis in QPM provides an energy-generating mechanism repressed in *o2*. This mechanism mollifies an energy deficiency, consistent with the previous assumption[30]. RILs generated after seven generations of self-pollination from a cross between K0326Y and W64A*o2*[16], together with the K0326Y genome sequence, will be valuable to evaluate the contribution of individual *Mo2*s to QPM phenotype.
”

As stated above, the discussion of PFP alpha, PPDK and chaperones should build off and cite results and interpretations previously published, not ignore them.

Response: Thanks for the comment. We have cited the paper and discussed this in the right place.

“Based on the previous studies of Holding et al.[30] and our findings, we propose a model for how starchy endosperm in *o2* is converted into a vitreous phenotype in QPM (Fig. 5). During endosperm development, zein proteins are stored in ER PBs, and their synthesis is influenced in a spatio-temporal pattern by ATP availability, which affects their numbers, sizes, and shapes. The inner maize endosperm is deficient in oxygen, and the glycoltic pathway, rather than the tricarboxylic acid (TCA) cycle and oxidative phosphorylation, becomes increasingly important to generate ATP (Supplementary Fig. 20). When normal zein PBs, functional ER, and normal starch biosynthesis occur simultaneously in the presence of sufficient ATP, it facilitates development of a vitreous endosperm[45]. However, the *o2* mutant significantly reduces the amount of zeins and appears to impair normal protein folding in the ER. It was hypothesized that the activation of the unfolded protein response (UPR) in *o2* requires increased amounts of energy in form of ATP and increased levels of chaperons, including heat shock proteins[30]. Moreover, the *o2* mutant has reduced expression of PPDK1, affecting glycolysis (Supplementary Fig. 20) and aggravating the energy shortage[31]. The *o2* mutation also reduces starch synthesis directly, resulting from transcriptional down-regulation of genes involved in its synthesis[41]. These metabolic changes impact the way in which PBs and starch grains congeal during kernel desiccation, leading to opacity in the mature endosperm[45]. Alternatively, the pleiotropic effects of *o2* mutations could be explained by additive effects from other processes. The *o2* effect on PB

structure through zein gene expression and ATP depletion, through *Pfpα* and *Ppdk1*, could be independent, but they combine to give a more severe endosperm phenotype than reducing zein proteins alone. This is consistent with multiple *Mo2*s involved in different metabolic pathways required to restore *o2* mutants to a vitreous phenotype (Fig. 5).
"

Line 287-289 "Noticeably, the inner maize endosperm environment is anoxic, and ATPs are generated mainly from glycolysis rather than the tricarboxylic acid (TCA) cycle (Supplementary Fig. 20)" This statement is based off theories offered in the previous 2012 plant physiology paper which cites the original work by Rolletschek et al that describes increasing hypoxia in the central endosperm. Neither paper has been cited here. It is not "noticeable". It has not been measured here and it cannot be stated that this leads to ATP coming mainly from glycolysis which is speculation but presented as fact.
Response: Response: Thanks for the comment. We have rephrased this part. The inner maize endosperm is deficient in oxygen, and the glycoltic pathway, rather than the tricarboxylic acid (TCA) cycle and oxidative phosphorylation, becomes increasingly important to generate ATP (Supplementary Fig. 20).

Line 289-293. Again this is speculative but presented as fact.
Response: We have rephrased it.

Line 293-294. This is not a novel idea. It was presented in the Plant Physiology paper and it is speculative.
Response: We have rephrased it.

Line 294-296. PPDK is a bi-directional glycolytic enzyme that can also act in the gluconeogenic direction. Therefore it is not possible to speculate on the effect of its reduced gene expression glycolytic flux or on ATP supply.
Response: We have made the description more specifically.
"Moreover, the *o2* mutant has reduced expression of PPDK1, affecting glycolysis (Supplementary Fig. 20) and aggravating the energy shortage[31]".

295-297. This is speculative and without reason about the effects of starch on vitreousness. All we know for sure is the effect of altered zein abundance and protein body number, shape and size on vitreousness.
Response: Thanks for the comment. We have rephrased and expanded this as follows:
"Moreover, the *o2* mutant has reduced expression of PPDK1, affecting glycolysis (Supplementary Fig. 20) and aggravating the energy shortage[31]. The *o2* mutation also reduces starch synthesis directly, resulting from transcriptional down-regulation of genes involved in its synthesis[41]. These metabolic changes impact the way in which PBs and starch grains congeal during kernel desiccation, leading to opacity in the mature endosperm[45]. Alternatively, the pleiotropic effects of *o2* mutations could be explained by additive effects from other processes. The *o2* effect on PB structure through zein gene expression and ATP depletion, through *Pfpα* and *Ppdk1*, could be independent, but they combine to give a more severe endosperm phenotype than reducing zein proteins alone. This is consistent with multiple *Mo2*s involved in different metabolic pathways required to restore *o2* mutants to a vitreous phenotype (Fig. 5)."

Line 306. No mention is made that PFPalpha is the regulatory subunit of a heterotetrameric holoenzyme in which PFP beta is the catalytic subunit.

Response: We have added the explanation in the right place.

"The PFP protein complex, consisting of two regulatory α-subunits and two catalytic β-subunits, is a non-ATP-dependent enzyme that could catalyze a rate-limiting step in glycolysis. It was proposed that increased PFPα activity in QPM could attenuate oxygen stress in the endosperm by increasing diminished ATP reserves; PFPβ expression was totally uniform between genotypes[30]."

Line 308-309 The idea that QPM uses an altered energy currency as part of the recovery of vitreous endosperm is not new but was extensively described before. Indeed, a curtailed description is presented which does not provide any new evidence for this theory beyond speculation

Response: Thanks for the comment. This is a genome manuscript first. We have added our evidences including gene expression data and the causal structural variations.

Line 309-311. "Partially restored ATP levels" This was not measured so this speculation is not valid.

Response: We have removed the statement.

The model presented ignores previous published data showing that the expression of many HSPs, that are elevated in o2, are returned to normal wild type expression levels in QPM endosperm and thus, that UPR is likely reduced in QPM endosperm compared with o2. The model presented implies that UPR stress is further increased in QPM endosperm which would be the opposite of what was previously shown.

Response: Thanks for highlighting the points. We added our explanation into text. HSPs, a group of chaperones that assist in protein folding and assembly, are affected by various abiotic and biotic stresses. The protective effects of HSPs help maintain optimal conditions for plant growth and development[43]. Our RNA-seq experiments show that most *Hsp* genes are expressed at a higher level in QPM compared to *o2* and wild types, indicating that the *o2* mutant may not effectively activate the heat shock response to cope with protein folding. HSP70 and HSP90 were shown to be greatly induced when root tissue was stressed[30]. Still, some down-regulated *Hsps* in QPM might have other roles due to functional divergence after maize tetraploidization. The effect on HSPs is reasonably consistent with earlier gene expression data that identified 8 *Hsps* among 37 down-regulated genes[30], compared with 49 (43 increased and 6 decreased) *Hsp* genes in 1791 DGEs in this study. Our increased resolution of gene expression might result from the longer 125-bp paired-end reads, increasing the ability to distinguish homologous *Hsp* members in two sets of maize sub-genomes, whereas previous study using the shorter 51-bp single-end reads and lower sequence output generated from the early Illumina platform had limited the power to define DGEs. A number of 43 incresed HSPs out of 49 indicated stonger UPRs in QPM. To make it clearer, we provided a detailed supplementary Table 19 and exphasized HSP genes in all DGEs in Supplementary Fig. 13.

Supplementary Table 19 | Differentially expressed genes associated with heat shock proteins and related genes that are common in two sets of vitreous QPMs and soft non-QPMs (K0326Y against W64A*o2*, CM105*Mo2* against CM105*o2*).

Gene ID	W64Ao2	K0326Y	Fold Change	p-value	CM105o2	CM105Mo2	Fold Change	p-value	Threshold	Chr	Start	End	Annotation
K0326Y_002415	8.4	0.7	-12.8	2.10E-04	55.1	10.5	-5.2	3.27E-06	Down	1	131971888	132000000	HSP 20
K0326Y_021553	24.5	10.7	-2.3	1.28E-04	148.0	66.6	-2.2	2.13E-07	Down	5	173239702	173000000	HSP 6 transcription factor
K0326Y_030319	34.9	22.4	-1.6	0.009340497	249.7	108.5	-2.3	1.26E-12	Down	10	74010456	74012914	DNAJ HSP
K0326Y_032860	20.4	1.2	-17.0	8.36E-09	38.6	10.2	-3.8	2.43E-05	Down	8	36444029	36445248	HSP 30
K0326Y_034083	74.8	32.3	-2.3	1.60E-05	53.9	20.6	-2.6	5.27E-05	Down	8	129266117	129000000	HSP 22
K0326Y_036433	361.0	232.0	-1.6	2.12E-11	371.6	145.5	-2.6	1.52E-32	Down	9	41673754	41675622	HSP binding protein
K0326Y_001009	7400.0	14607.7	2.0	2.33E-297	3521.0	57005.6	16.2	0.00E+00	Up	1	38700000	38724325	HSP 10
K0326Y_002528	7010.5	12532.5	1.8	1.53E-187	4217.4	9613.4	2.3	0.00E+00	Up	1	163965128	163968703	HSP 70
K0326Y_003023	45.6	1154.2	25.3	4.31E-92	28.0	2600.4	92.7	8.65E-144	Up	1	191000000	191043092	HSP 8
K0326Y_003024	5362.3	25589.7	4.8	0.00E+00	309.0	9739.7	31.5	0.00E+00	Up	1	191295608	191299465	HSP 90
K0326Y_003274	54.6	1470.4	26.9	2.08E-252	41.3	862.9	20.9	8.68E-154	Up	1	202595093	202596715	HSP 8 transcription factor
K0326Y_003276	147.0	2024.2	13.8	0.00E+00	121.0	2804.5	23.2	0.00E+00	Up	1	202721686	202725075	Activator of HSP 90
K0326Y_004210	587.2	3535.3	6.0	0	123.0	4132.9	33.6	0	Up	1	246909282	246912963	HSP 24 transcription factor
K0326Y_004728	4295.5	8597.4	2.0	1.17E-194	2025.8	13997.5	6.9	0.00E+00	Up	1	271206392	271220319	HSP 70
K0326Y_005002	111.0	3319.6	29.9	0.00E+00	27.7	3745.5	135.2	0.00E+00	Up	1	283153135	283155553	HSP 17 transcription factor
K0326Y_005336	47.1	252.6	5.4	4.83E-50	120.4	669.6	5.6	8.19E-67	Up	1	295000000	295000000	HSP 11 transcription factor
K0326Y_008759	161.0	896.0	5.6	2.72E-125	180.0	416.8	2.3	7.19E-26	Up	2	191902640	191911518	HSP 18 transcription factor
K0326Y_008822	5104.7	10946.7	2.1	0	1863.9	8540.5	4.6	0.00E+00	Up	2	194748165	194751940	HSP 90
K0326Y_008855	1869.6	3082.3	1.6	3.14E-71	1500.6	4075.2	2.7	1.43E-246	Up	2	195329352	195332656	HSP 70
K0326Y_009837	14.1	57.2	4.1	3.45E-12	1.7	150.8	89.9	8.03E-21	Up	2	229000000	229119029	HSP 22
K0326Y_011022	1.0	91.1	94.3	1.08E-16	0.3	60.8	182.4	5.15E-09	Up	3	23400000	23363609	HSP 12
K0326Y_011039	0.3	34.6	127.8	1.47E-08	5.0	614.6	122.4	4.56E-67	Up	3	23490079	23500000	HSP 17
K0326Y_011851	8336.4	13883.9	1.7	5.71E-136	9209.0	15565.1	1.7	1.32E-138	Up	3	103782284	103792129	HSP 70
K0326Y_013912	379.0	2198.2	5.8	3.31E-197	50.3	222.6	4.4	3.67E-44	Up	3	222136122	222148796	HSP 90
K0326Y_015165	48.8	450.6	9.2	1.94E-134	18.4	1418.6	77.1	3.73E-199	Up	4	55094422	55097851	Activator of HSP 90
K0326Y_016200	2109.8	4028.7	1.9	6.77E-95	891.7	4528.5	5.1	0.00E+00	Up	4	146006483	146014683	HSP70
K0326Y_016947	3730.0	21402.6	5.7	0.00E+00	297.9	25916.6	87.0	0.00E+00	Up	4	181550391	181551586	HSP 22
K0326Y_017403	1540.8	3020.7	2.0	4.15E-63	1418.1	4139.3	2.9	1.69E-149	Up	4	198737669	198742128	HSP 90
K0326Y_017891	26.8	143.4	5.4	9.91E-28	5.9	137.4	23.1	5.63E-31	Up	4	226197710	226203029	HSP 101
K0326Y_018062	1088.5	5280.0	4.9	0	173.8	4762.4	27.4	0	Up	4	229963598	229965758	HSP 40
K0326Y_019190	3732.3	6376.7	1.7	3.87E-101	695.1	9595.2	13.8	0.00E+00	Up	5	12591095	12600000	HSP 70
K0326Y_021239	4.8	27.3	5.7	2.59E-09	55.3	108.5	2.0	3.22E-04	Up	5	154203643	154205141	HSP 18 transcription factor
K0326Y_022095	2143.4	3907.8	1.8	1.22E-132	605.4	4008.8	6.6	0.00E+00	Up	5	195365858	195372177	HSP70
K0326Y_022779	19.7	423.0	21.5	2.33E-29	3.0	507.0	170.4	3.40E-34	Up	5	221840104	221841162	HSP 17
K0326Y_025006	1470.0	4609.3	3.1	0.0371	0.6	21.8	34.4	0.000238	Up	6	122000000	121660566	HSP 18
K0326Y_025191	626.8	3305.8	5.3	0.00E+00	454.9	6660.3	14.6	0.00E+00	Up	6	130513831	130519849	HSP 70
K0326Y_026055	27.3	99.7	3.7	1.51E-16	131.9	199.6	1.5	4.07E-03	Up	6	172619513	173000000	HSP 22
K0326Y_026209	733.0	3690.6	5.0	0.00E+00	1765.2	3462.6	2.0	1.37E-129	Up	7	1731363	1730000	HSP 3
K0326Y_028042	1443.9	9537.2	6.6	0.00E+00	619.4	11485.3	18.5	0.00E+00	Up	7	138042122	138046965	HSP 90
K0326Y_028084	1722.9	4484.0	2.6	0.00E+00	849.2	11923.6	14.0	0.00E+00	Up	7	139972693	139976246	HSP 4
K0326Y_030597	349.8	3005.2	8.6	0	126.1	24953.4	197.9	0.00E+00	Up	10	91568371	91571136	HSP 90
K0326Y_031035	1.3	6.2	4.8	2.29E-03	24.5	1115.3	45.5	2.70E-145	Up	10	119000000	119000000	HSP 22
K0326Y_031520	14.1	684.8	48.6	3.67E-41	12.3	959.1	78.0	4.79E-46	Up	10	136514515	136515760	HSP 20 transcription factor
K0326Y_032526	541.0	11212.5	20.7	1.16E-27	214.7	9725.0	45.3	3.50E-42	Up	8	22600000	22556483	HSP 18
K0326Y_033259	148.3	921.0	6.2	3.94E-191	55.2	2814.3	51.0	0.00E+00	Up	8	74673581	74675640	HSP 40
K0326Y_033975	3639.9	5844.4	1.6	2.06E-72	173.1	11589.0	67.0	0.00E+00	Up	8	123264071	123266896	HSP 6
K0326Y_035268	14121.7	31973.4	2.3	0.00E+00	5641.3	93185.7	16.5	0.00E+00	Up	8	175126442	175129341	HSP 1
K0326Y_035804	520.0	2975.3	5.7	0.00E+00	1181.8	4422.6	3.7	0.00E+00	Up	9	11300000	11254812	HSP 24
K0326Y_038055	330.1	1502.7	4.6	7.53E-211	45.4	3958.2	87.1	0	Up	9	146825264	146829347	HSP 70

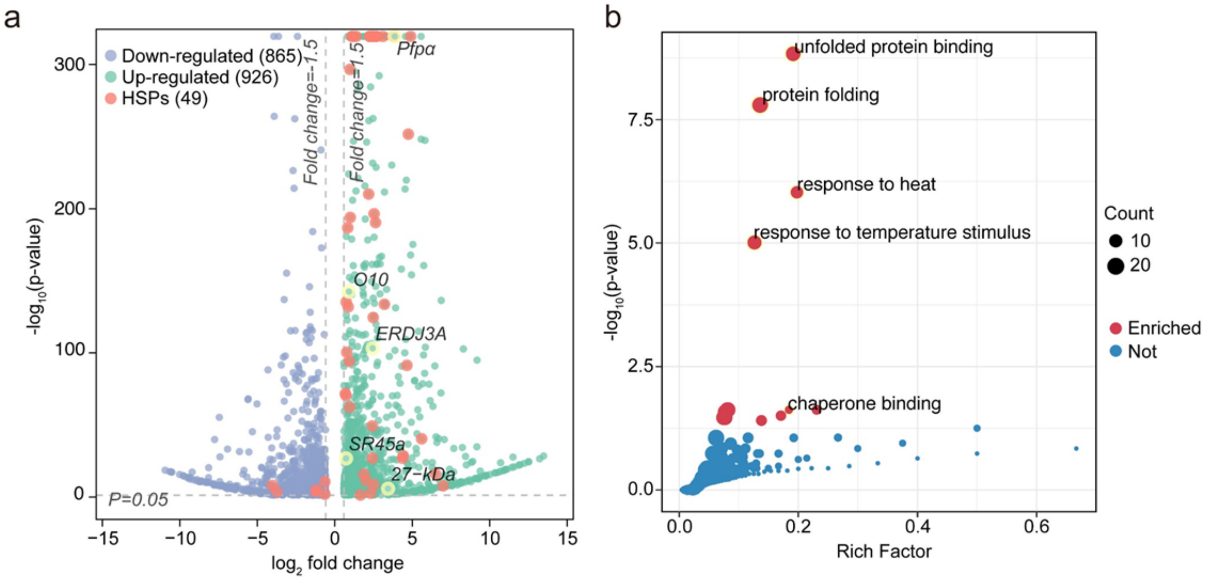

Supplementary Fig. 13 | Differential expression of genes and GO enrichment between QPM and *o2* maize. (a) Volcano plot of differentially expressed gene between two sets of vitreous QPMs and soft non-QPMs (K0326Y against W64A*o2*, CM105*Mo2* against CM105*o2*). The up-regulated genes were shown in green and the down-regulated genes were illustrated in light purple. The heat shock related proteins (HSPs) were shown in orange. (b) Gene ontology (GO) enrichment analysis of for up-regulated genes in QPM maize. The Rich Factor was the ratio of differentially expressed gene numbers against all annotated gene numbers.

REVIEWERS' COMMENTS:

Reviewer #1 (Remarks to the Author):

Comments for the manuscript entitled “Long-read sequencing reveals genomic structural variations that underlie creation of Quality Protein Maize”

The authors addressed the questions I asked to some extent, but I do not think the manuscript has been significantly improved. The section of cloning Mo2S is still full of speculations. In addition, the manuscript needs to be more concise and better structured. It was difficult to find a logic frame and key points through the manuscript. For example,

1. Line 51-54. This is part of the introduction, but it looks like a result.

2. Line 83-87. This sentence should be in the result section as well.

There are many issues like this in the manuscript, but I am not pointing out them one by one. The authors should do their best and find ways to improve the manuscript by themselves before submitting it to a journal. There is some good advice on the webpage (https://www.nature.com/articles/d41586-018-02404-4). I hope they are useful to the authors. For this specific manuscript, these are something I would do.

1. Write down the logic flow.
2. Restructure the whole manuscript.
3. Remake figures and tables if necessary
4. Reduce to word count from 5200 to 3000.
5. Take good care of the language.

Reviewer #2 (Remarks to the Author):

Along with extensive minor things needing attention, there were two major criticisms of the original submission. First, the paper failed to adequately discuss the significance of the sequencing of a QPM genome in terms of its advances over previously published maize genomes and its specific significance for studying and further developing Quality Protein Maize. Second, the discussion was overly speculative about the role of candidate genes and in several instances, made statements that were not supported by the experimental data. Even more significantly, the authors did not put the new data into the context of previously existing data and previous theories concerning its significance.
I am happy to see that all of the above shortfalls have been carefully corrected in the revised submission and the result is a substantially better and highly significant manuscript.
David Holding

REVIEWERS' COMMENTS:

Reviewer #1 (Remarks to the Author):

Comments for the manuscript entitled “Long-read sequencing reveals genomic structural variations that underlie creation of Quality Protein Maize”

The authors addressed the questions I asked to some extent, but I do not think the manuscript has been significantly improved. The section of cloning Mo2S is still full of speculations. In addition, the manuscript needs to be more concise and better structured. It was difficult to find a logic frame and key points through the manuscript. For example,

1. Line 51-54. This is part of the introduction, but it looks like a result.
Response: Thanks for the comment. We totally agree. We have removed it since we have such information in the results.
2. Line 83-87. This sentence should be in the result section as well.
Response: The nice suggestion was taken. We deleted the details and summarized our results in a very concise way in the Introduction.
There are many issues like this in the manuscript, but I am not pointing out them one by one. The authors should do their best and find ways to improve the manuscript by themselves before submitting it to a journal. There is some good advice on the webpage (https://www.nature.com/articles/d41586-018-02404-4). I hope they are useful to the authors. For this specific manuscript, these are something I would do.
1. Write down the logic flow.
2. Restructure the whole manuscript.
3. Remake figures and tables if necessary
4. Reduce to word count from 5200 to 3000.
5. Take good care of the language.
Response: Thank you for your review and comments in terms of the content structure and language. The comments are valuable and very critical to improve our manuscript. We re-drafted our manuscript in a more logic flow and revised our manuscript accordingly. We modified our figures and tables concerning the color, format, error bars, scale bars and other details. We downsized the main text into 4796 words. The co-author Dr. Brian Larkins, who is a native English speaker has carefully polished the language again.

Reviewer #2 (Remarks to the Author):

Along with extensive minor things needing attention, there were two major criticisms of the original submission. First, the paper failed to adequately discuss the significance of the sequencing of a QPM genome in terms of its advances over previously published maize genomes and its specific significance for studying and further developing Quality Protein Maize. Second, the discussion was overly speculative about the role of candidate genes and in several instances, made statements that were not supported by the experimental data. Even more

significantly, the authors did not put the new data into the context of previously existing data and previous theories concerning its significance.

I am happy to see that all of the above shortfalls have been carefully corrected in the revised submission and the result is a substantially better and highly significant manuscript.

Response: We appreciate the reviewer’s great suggestions and comments, which greatly improved our manuscript.
